# Small molecule-mediated up-regulation of microRNA targeting a key cell death modulator BNIP3 improves cardiac function following ischemic injury

**DOI:** 10.1038/srep23472

**Published:** 2016-03-24

**Authors:** Se-Yeon Lee, Seahyoung Lee, Eunhyun Choi, Onju Ham, Chang Youn Lee, Jiyun Lee, Hyang-Hee Seo, Min-Ji Cha, Bohyun Mun, Yunmi Lee, Cheesoon Yoon, Ki-Chul Hwang

**Affiliations:** 1 Brain Korea 21 PLUS Project for Medical Science, Yonsei University College of Medicine, Seoul 120-752, Republic of Korea; 2 Department of Biomedical Sciences, College of Medicine, Catholic Kwandong University, Gangneung, Gangwon-do 210-701, Republic of Korea; 3 Department of Integrated Omics for Biomedical Sciences, Graduate School, Yonsei University, Seoul 120-749, Republic of Korea; 4 Department of Chemistry, Kwangwoon University, Seoul 01897, Republic of Korea; 5 Department of Cardiovascular & Thoracic Surgery, College of Medicine, Catholic Kwandong University, Gangneung, Gangwon-do 210-701, Republic of Korea

## Abstract

Genetic ablation of BCL2/adenovirus E1B 19 kDa protein-interacting protein 3 (BNIP3), an essential regulator of cardiac cell death, is an effective way to prevent cardiac cell death triggered by pathologic conditions. However, currently there exists no known means, such as inhibitors, to down-regulate BNIP3 in mature heart. Here, we report that a small molecule inducer of microRNA-182 (miR-182) suppressed ischemia/reperfusion (I/R)-induced cardiac cell death by down-regulating BNIP3. We first selected miR-182 as a potent BNIP3-targeting miRNA based on miRNA-target prediction databases and empirical data. The subsequent screening of small molecules for inducing miR-182 expression identified Kenpaullone as a hit compound. *B*oth exogenous miR-182 and Kenpaullone significantly suppressed hypoxia-induced cardiomyocyte death *in vitro*. To investigate the effect of changing substituents of Kenpaullone on miR-182 expression, we synthesized 9 derivatives of Kenpaullone. Among these derivatives, compound **5** showed significantly improved ability to induce miR-182 expression. The results of the *in vivo* study showed that compound **5** significantly improved heart function following I/R-injury in rats. Our study provides strong evidence that the small molecule-mediated up-regulation of miRNAs is a viable strategy to down-regulate target proteins with no known chemical inhibitor and that compound **5** may have potential to prevent I/R-inflicted cardiac cell death.

The ubiquitous death protein BCL2/adenovirus E1B 19 kDa protein-interacting protein 3 (BNIP3) is involved in various types of cardiac cell death - namely necrosis, autophagy, and apoptosis - under hypoxic condition^1^,^2^,^3^. Although BNIP3-dependent autophagosome-mediated clearance of damaged mitochondria could be cyto-protective^4^, it was not strong enough to prevent the cardiac cell death inflicted by ischemia-reperfusion (I/R) injury^5^. On the other hand, genetic ablation of BNIP3 prevented cardiomyocyte apoptosis^6^, suggesting that suppression of BNIP3 may be an effective therapeutic approach to prevent and/or minimize the cardiac cell death. Nevertheless, the genetic ablation of BNIP3 is not a currently feasible option for treating mature heart and there are no known inhibitors of BNIP3 available. Thus, finding innovative means to regulate the expression of BNIP3 *in vivo* may provide us valuable tools for managing ischemic heart disease which has been a leading cause of death worldwide for many decades^7^.

One of the means to down-regulate proteins of interest is by utilizing microRNAs (miRNAs). MicroRNAs are highly conserved non-coding short RNAs that negatively regulate target gene expressions by either inhibiting translation or degrading target mRNAs, functioning as endogenous inhibitors of targeted proteins. This characteristic makes miRNAs a promising candidate for facilitating targeted down-regulation of BNIP3 expression *in vivo*. Regarding miRNAs targeting BNIP3, it has been reported that miR-210 and miR-145 exerted anti-apoptotic effect by targeting BNIP3^8^,^9^. Down-regulation of BNIP3 by miR-210 protected neural progenitor cells from apoptosis^8^, and miR-145 protected cardiomyocytes from hydrogen peroxide (H_2_O_2_)-induced apoptosis by suppressing BNIP3^9^. These indications correlate with our hypothesis.

Although there is also a previous report suggesting that BNIP3 transcriptionally represses apoptosis-inducing factor (AIF) so that the miR-145-mediated down-regulation of BNIP3 consequently results in the up-regulation of AIF and subsequent death of prostate cancer cells^10^, care must be taken in interpreting such results since there are numerous oncogenic genes, as well as related signaling pathways, that have been confirmed as targets of miR-145^11^. Therefore, it is possible that the observed tumor-suppressing (or pro-apoptotic) effects of miR-145 in the latter study mentioned may have involved the down-regulation of targets other than BNIP3.

Collectively, these studies support our hypothesis that the attenuation of BNIP3 expression under pathologic conditions may protect cells from cell death. However, *in vivo* delivery of miRNA has unsolved technical issues such as low cellular uptake and instability in serum that may compromise the therapeutic efficacy of miRNA treatment^12^. Therefore, we exploited the induction of endogenous microRNAs (miRNA) by small molecules to down-regulate the expression of BNIP3 as a contingency strategy. The expert consensus is that both small molecule and miRNA are promising chemistry-based modalities for cardiac regeneration^13^, and the present study could be an effective seminal study to examine their therapeutic utility.

In this study, we first identified miR-182 as a miRNA that may effectively down-regulates BNIP3 expression in cardiomyocytes based on miRNA-target prediction algorithms and empirical data. Previous studies have indicated that miR-182 plays a significant role in cancer cell proliferation and invasion, and its known targets include N-myc downstream regulated gene (NDRG)^14^, programmed cell death 4 (PDCD4)^15^, special AT-rich sequence-binding protein 2 (SATB2)^16^, and number of cancer metastasis-related genes^17^. Additionally, miR-182 has been implicated in sensory organ development^18^ and skeletogenesis^19^. However, except the reported miR-182-mediated down-regulation of Rac1^20^, not much is currently known about the role and specific targets of miR-182 in cardiomyocytes.

After identifying miR-182 as a BNIP3-targeting miRNA in cardiomyocytes, we further screened a range of small molecules to find a chemical inducer of endogenous miR-182. We examined the effects of selected small molecules on the induction of endogenous miR-182 expression and subsequent BNIP3 expression in cardiomyocytes. Additionally, we synthesized 9 derivatives of the selected small molecule to investigate whether varying substituents could enhance the anti-cell death effect of the selected base small molecule. Among those derivatives, compound **5** showed significantly enhanced ability to induce miR-182 expression and it also significantly improved heart function following I/R-injury in rats. Our study provides strong evidence that the small molecule-mediated up-regulation of miRNAs is a viable strategy to down-regulate target proteins with no known chemical inhibitor and that compound **5** may have potential to prevent I/R-inflicted cardiac cell death.

## Results and Discussion

### Expressions of cell death markers and BNIP3 in I/R injured heart

To establish that BNIP3 plays a prominent role in cell death, the expressions of different cell death markers–namely, cyclophillin D (necrosis), LC3A/B (autophagy), and caspase 3 (apoptosis)–in I/R injured heart were examined by immunohistochemistry. Our data demonstrated that I/R injury to heart inflicted all three types of cell death and that BNIP3 was concomitantly expressed with those markers of various types of cell death (Supplementary Fig. 1A). Furthermore, I/R injury to heart decreased the expression of Bcl-2 (B-cell lymphoma 2), an anti-apoptotic protein, while it increased the expressions of both pro-apoptotic members of the Bcl-2 family Bak (Bcl-2 homologous antagonist killer) and BNIP3 (Supplementary Fig. 1B). These data suggest that BNIP3 is involved in I/R injury-induced cardiac cell death as previously reported^3^.

### Screening of miRNAs targeting BNIP3

**Figure 1:**
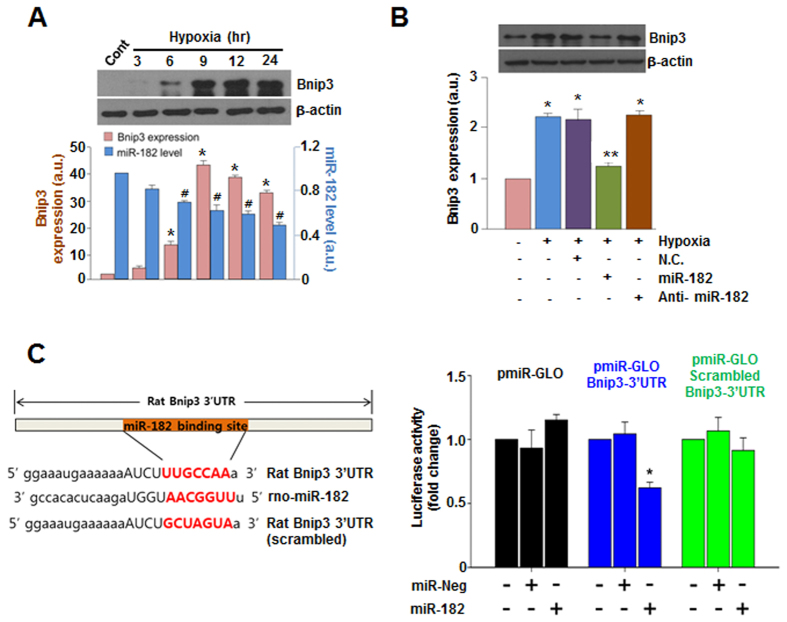
BNIP3 is a direct target of miR-182. (**A**) Time-dependent expression changes of BNIP3 under hypoxia. The expression of BNIP3 and miR-182 were compared at matching time points. *p < 0.05 compared to control. ^#^p < 0.05 compared to control. (**B**) Effect of anti-miR-182 on miR-182-induced down regulation of BNIP3. *p < 0.05 compared to normal control. **p < 0.05 compared to hypoxia only. (**C**) Luciferase assay using 3′UTR of BNIP3. miR-Neg: negative control miRNA. *p < 0.05. The luciferase activity of the cells only transfected with each vector served as controls for each vector group with relative value of 1.

To select miRNAs that could down-regulate BNIP3 expression, we had thoroughly searched and compared multiple miRNA databases such as TargetScan (www.targetscan.org)^21^ and miRBase (www.mirbase.org)^22^, and subsequently chose 8 miRNAs that potentially target BNIP3 (Supplementary Fig. 2). Among these 8 miRNA candidates, miR-182 most effectively suppressed the hypoxia-induced increase in BNIP3 expression per results of mimic treatment (Supplementary Fig. 3). Furthermore, we observed that the expression levels of BNIP3 and miR-182 under hypoxic conditions were inversely proportional to each other (Fig. 1A), indicating that BNIP3 may be a target for miR-182 in cardiomyocytes. Anti-miR-182 alone did not further increase the expression of BNIP3 in cardiomyocytes under hypoxic conditions (Fig. 1B), suggesting that the expression level of miR-182 may have been too low to be significant. Additionally, we examined the effect of increasing concentrations of anti-miR-182 (20, 50, and 100 nM) on the presumably miR-182-mediated down-regulation of BNIP3 expression in cardiomyocytes under hypoxic conditions. When 100 nM of anti-miR-182 was co-delivered with miR-182 (100 nM), BNIP3 expression was recovered (Supplementary Fig. 4). This indicates that 100 nM may be sufficient to abrogate the effects of the delivered exogenous miR-182; it also indirectly shows that the observed down-regulation of BNIP3 with miR-182 was indeed mediated by miR-182. To further demonstrate that miR-182 targets the 3′UTR region of BNIP3 mRNA, we utilized luciferase reporter constructs containing wild or scrambled miR-182 binding sites of the BNIP3 3′UTR. Transfection of miR-182 suppressed the expression of luciferase in cells with the wild type miR-182 binding site, but not in cells with the scrambled miR-182 binding site. These data indicate that miR-182 targets the 3′UTR region of BNIP3 mRNA in a sequence-specific manner (Fig. 1C).

### miR-182 suppresses hypoxia-induced BNIP3 expression and apoptotic events

**Figure 2:**
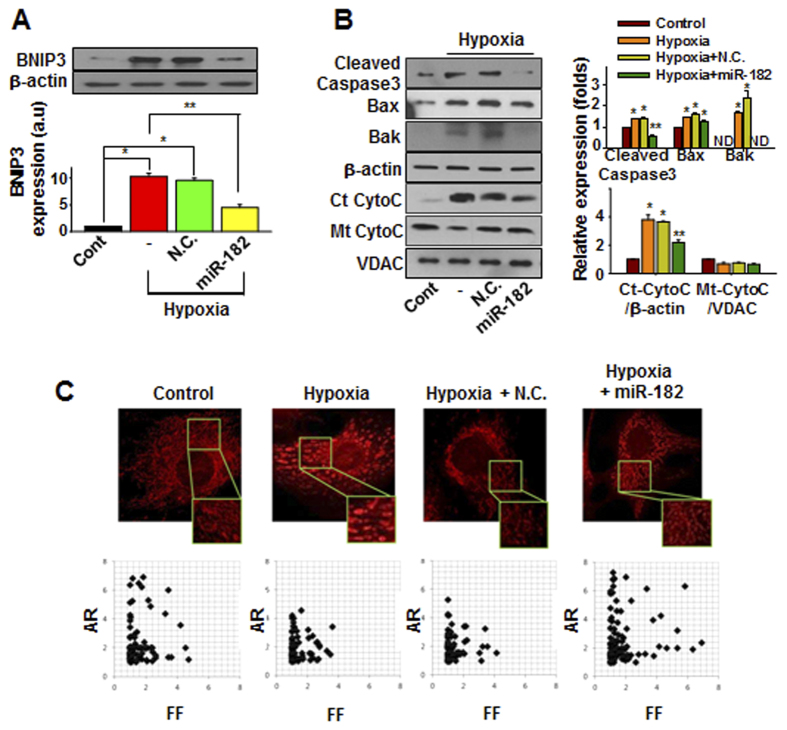
miR-182 suppresses hypoxia-induced apoptotic events. (**A**) The expression of BNIP3 under hypoxia with or without miR-182 transfection was evaluated by western blot. N.C. (negative control miRNA). *p < 0.05 compared to control. **p < 0.05 compared to hypoxia group. (**B**) The expression of apoptosis-related genes under hypoxia with or without miR-182 transfection. Ct-CytoC: cytosolic cytochrome C; Mt-CytoC: mitochondrial cytochrome C. *p < 0.05 compared with control. **p < 0.05 compared with hypoxia group. (**C**) Cells were transfected with either negative control (N.C.) miRNA or miR-182 prior to hypoxia treatment. After 24 hours of hypoxia, the cells were loaded with MitoTracker dye and images were taken by using a confocal microscopy. Form factor (FF, the reciprocal of circularity value) and aspect ratio (AR, the ratio between the major and minor axis of an ellipse equivalent to the object) were calculated.

Treatment with the miR-182 mimic significantly mitigated several hypoxia-induced effects, including the suppression of BNIP3 expression (Fig. 2A), the reduction of pro-apoptotic signaling (Fig. 2B), and the attenuation of mitochondrial fission (Fig. 2C), each of which is typically observed in the setting of BNIP3-induced apoptosis^23^. These data demonstrate that the down-regulation of BNIP3 via the exogenous delivery of miR-182 mimics effectively prevents the activation of pro-apoptotic processes in cardiomyocytes under hypoxic conditions.

### Screening of small molecule inducer of miR-182

**Figure 3:**
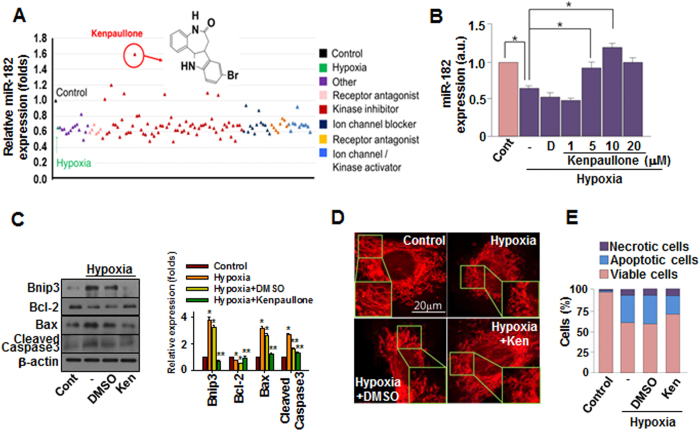
miR-182 inducing small molecule Kenpaullone suppresses hypoxia-induced apoptosis. (**A**) Screening of in-house small molecule library for inducing miR-182. Cells were treated with 10 μM of each small molecule for 24 hours. Relative miR-182 expression was measured by real-time PCR. (**B**) Cells were exposed to hypoxia with varying concentrations of kenpaullone treatment. miR-182 expression was measured by real-time PCR. D: DMSO. *p < 0.05 (**C**) The expression of BNIP3 and apoptosis-related genes under hypoxia with or without kenpaullone (Ken, 10μM) treatment was evaluated by western blot analysis. (**D**) Representative images of mitochondrial fission induced by hypoxia with or without kenpaullone treatement. (**E**) Cellular apoptosis was evaluated by flow cytometry. Ken: kenpaullone.

Although the use of either exogenous miRNAs or small interfering RNAs (siRNAs) for therapeutic purposes is theoretically viable and has yielded encouraging results^24^,^25^, critical issues such as their low cellular uptake, off-target effects, and instability in serum must be addressed before miRNA-based therapeutics can be implemented^12^. As an alternative to delivering exogenous therapeutic miRNAs, we have utilized a small molecule-based approach to intensify the expression of specific endogenous miRNAs. Small molecule-mediated modulation (inhibition or activation) of miRNAs is a recent approach which utilizes RNA interference to accomplish sequence-specific silencing of target genes for therapeutic purposes^26^. Only a handful of studies had reported small molecule-mediated modulation of specific miRNAs^27^,^28^,^29^, making effort to identify miRNA-modulating small molecules therapeutically relevant. We screened our in-house chemical library, including receptor agonists and antagonists, kinase inhibitors, and ion channel activators and inhibitors, for the ability to increase miR-182 expression in hypoxic cardiomyocytes. Our approach contrasts from those utilized by the majority of recent studies involving small molecule-mediated miRNA regulation, as those studies focused on the negative regulation of miRNAs with small molecules^30^,^31^. Among the small molecules screened for their ability to induce miR-182 expression under hypoxic conditions, kenpaullone increased the expression of miR-182 most significantly (Fig. 3A) and did so in a concentration-dependent manner up to 10 μM (Fig. 3B).

### Small molecule kenpaullone attenuates hypoxia-induced apoptosis

Treatment with kenpaullone down-regulated BNIP3 expression dramatically and also attenuated the induction of pro-apoptotic molecules, such as Bax and cleaved caspase 3 (Fig. 3C). Kenpaullone effectively prevented hypoxia-induced mitochondrial fission (Fig. 3D and Supplementary Fig. 5A) and apoptosis (Fig. 3E and Supplementary Fig. 5B). Furthermore, the hypoxia-induced production of reactive oxygen species (ROS), which exacerbate cellular apoptosis^32^, was significantly decreased by kenpaullone treatment (Supplementary Fig. 6). Together, these data indicate that kenpaullone is a small molecular inducer of miR-182 which exerts anti-apoptotic effects on cardiomyocytes exposed to hypoxia.

### Synthesis of kenpaullone derivatives and their effect on miR-182 and BNIP3 expression

To examine whether changing the substituents of kenpaullone would enhance the ability to induce miR-182 expression, we synthesized 9 derivatives, which were derived from the Fischer indole reactions of 3,4-dihydro-1H-benzo[b]azepine-2,5-dione 11 with various substituted phenylhydrazines under acidic conditions^33^,^34^. (Supplementary Fig. 7A and see General procedures in the Supporting Information Chemical). Out of 10 compounds (Kenpaullone and 9 derivatives) tested, the compound **5** having a nitro substituent was most effective in inducing miR-182 expression (Supplementary Fig. 7B), and the effect was concentration dependent (Supplementary Fig. 7C). Compound **5** also significantly suppressed hypoxia-induced BNIP3 expression (Supplementary Fig. 8A). Additionally, anti-miR-182 (50 nM) attenuated compound **5-**mediated BNIP3 down-regulation (Supplementary Fig. 8B). Treatment with compound **5** alone or co-treatment with compound **5** and miR-182 (100 nM) completely suppressed BNIP3 expression; however, when high concentrations of anti-miR-182 (100 nM) were co-delivered with compound **5** and miR-182 (100 nM), BNIP3 expression was detectable (Supplementary Fig. 8C). These data indicate that the observed decrease of BNIP3 was indeed mediated by (**5**-induced) miR-182.

### Compound 5 induced miR-182 expression and subsequent BNIP3 down-regulation is independent of β-catenin pathway

Kenpaullone is an inhibitor of glycogen synthase kinase 3β (GSK3β)^35^ and inhibition of GSK3β results in the activation of β-catenin^36^, a transcriptional co-factor for Wnt-signaling target genes. Additionally, it has been reported that β-catenin induced miR-182 expression in breast cancer^37^. Thus, we examined whether compound **5**-mediated increase of miR-182 expression was β-catenin-dependent by using a β-catenin inhibitor (FH535)^38^. Pre-treatment with FH535 failed to prevent the compound **5**-induced increase of miR-182 under hypoxic conditions (Supplementary Fig. 9A). Furthermore, compound **5**-mediated down-regulation of BNIP3 under hypoxic conditions was not reversed by FH535 pre-treatment (Supplementary Fig. 9B), indicating that the classical GSK3β/β-catenin pathway is not the major mechanism underlying miR-182 induction. Although we were unable to identify the transcription factor by which compound **5** up-regulates miR-182 expression in cardiomyocytes in the present study, additional transcriptional factors are currently being examined in our laboratory as potential pivotal factors in this process. An example is hypoxia inducible factor 1 alpha (HIFα). It has been reported that miR-182 is under the transcriptional regulation of HIFα^39^, and inhibition of GSK3β resulted in decreased proteasomal destruction of HIF1α^40^, suggesting possible involvement of HIF1α in the compound **5**-induced up-regulation of miR-182. Another candidate transcription factor is signal transducer and activator of transcription 5 (STAT5) that has been reported to facilitate interleukin 2 (IL-2)-induced up-regulation of miR-182 in helper T lymphocytes^41^. The results of the ongoing investigation on the other transcription factors, including but not limited to, HIF1α and STAT5, will provide more clues on the underlying mechanisms of compound **5**-mediated expression of miR-182 in further studies.

### Effect of miR-182 and compound 5 on cardiac cell death *in vitro* and *in vivo*

**Figure 4:**
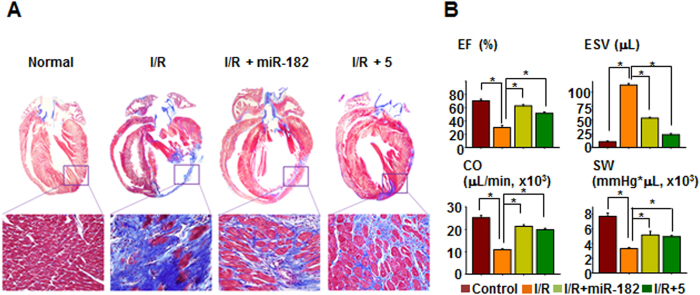
Compound 5 prevents cardiac fibrosis and improves heart function. (**A**) Cardiac fibrosis was evaluated by Trichrome staining 3 weeks after I/R-injury. (**B**) Cardiac function analysis. EF: ejection fraction; ESV: end systolic volume; CO: cardiac output; SW: stroke work. *p < 0.05.

To examine the effect of compound **5** on cardiomyocyte apoptosis *in vitro*, the cells were exposed to hypoxia with treatment of compound **5**, miR-182, anti-miR-182, or their combinations (Supplementary Fig. 10). Treatment with compound **5** or compound **5** and miR-182 attenuated hypoxia-induced apoptosis of cardiomyocytes, while the addition of anti-miR-182 diminished the anti-apoptotic effect of compound **5** and miR-182. To further verify the anti-cell death effects of miR-182 and compound **5** *in vivo*, we utilized a rat heart I/R injury model. When either the miR-182 mimic (locally applied to the border zone in gel) or compound **5 (**systemically delivered via intravenous injection) was applied immediately following I/R injury, various markers of cell death (necrosis, autophagy, and apoptosis) and the expression of BNIP3 were suppressed in the I/R-injured heart (Supplementary Fig. 11A). The number of terminal deoxynucleotidyl transferase dUTP nick end labeling (TUNEL)-positive cells was decreased in both the miR-182- and the compound **5**-treated I/R group compared to the control I/R group (Supplementary Fig. 11B). The immunohistochemical staining of CD31, a well-known endothelial cell marker, demonstrated that miR-182 and compound **5** increased the number of capillaries in the heart. Moreover, the expression of ventricular myosin light chain-2 (MLC-2v), a key regulator of cardiac muscle contraction^42^,^43^, was relatively well-preserved by both miR-182 and compound 5 treatment (Supplementary Fig. 12). Connexin 43 (Cx43) plays an important role in intercellular electrical and metabolic coupling, as well as cytoprotection^44^, and the expression of Cx43 was increased by both miR-182 and compound **5** (Supplementary Fig. 13). Trichrome staining of the heart demonstrated that both miR-182 and compound **5** significantly attenuated cardiac fibrosis (Fig. 4A). In addition, the parameters of cardiac function, such as ejection fraction (EF) and end-systolic volume (ESV), were significantly improved by both miR-182 and compound **5** compared to the control I/R group (Fig. 4B). Altogether, these data strongly suggest that the improved survival of cardiomyocytes via miR-182 and compound **5** treatment may have resulted in improved cardiac function following I/R injury.

In conclusion, our study demonstrated that the miRNA-mediated down-regulation of BNIP3 protected cardiac cells from cell death inflicted by I/R injury. Furthermore, we have provided strong evidence that the small molecule-mediated induction of endogenous miRNA *in situ* represents a viable and potent therapeutic approach to modulate key pathogenic factors. Our new approach to regulate endogenous miRNAs of therapeutic capability will broaden the field of both miRNA- and small molecule-based therapeutic research and may provide clinicians with improved means to treat cardiovascular disease.

## Methods

### Isolation neonatal rat ventricular cardiomyocytes

All experimental procedures for animal studies were approved by the Committee for the Care and Use of Laboratory Animals, Yonsei University College of Medicine, and were performed in accordance with the committee’s guidelines and regulations for animal care (2012-0198-3). One- to two-day-old Sprague-Dawley rat pups were anesthetized via hypothermia. In brief, pups were placed in a latex glove and immersed up to the neck in ice for 5–8 min to reach a surgical plane of anesthesia. This induction method lasts approximately 10 min. Then, the hearts were extracted and washed in Dulbecco’s phosphate-buffered saline solution (pH 7.4, Gibco BRL, USA) lacking Ca^2+^ and Mg^2+^. The hearts were minced into many pieces of approximately 1 mm3 and enzymatically digested with 10 ml of collagenase II (0.8 mg/ml, 262 units/mg, Gibco BRL) for 5 min in a 37 °C incubator. The supernatant was removed, and the minced tissue was treated with a fresh collagenase II solution for an additional 7 min in a 37 °C incubator. The supernatant was collected and diluted with cell culture medium (α-MEM containing 10% fetal bovine serum, Gibco BRL). Then, supernatant was collected with cell culture medium transferred to a new tube. The tubes were centrifuged at 1,800 rpm for 3 min at room temperature, and the cell pellets were resuspended with 2 ml of cell culture medium. The above procedures were repeated 7–9 times until little tissue remained. The cell suspensions were collected and seeded in 100 mm tissue culture dishes for 4–6 hr to reduce fibroblast contamination. The non-adherent cells were collected and seeded to achieve a final concentration of 5 × 10^5^ cells/ml. After 4–6 hr of incubation, the cells were rinsed twice with cell culture medium, and 0.1 μmol/L 5-bromo-2′-deoxyuridine (BrdU) was added. BrdU supplementation is recommended to increase the purity of the cardiomyocytes. The cells were then cultured with 10% (v/v) FBS in a CO_2_ incubator at 37 °C.

### Mitochondrial morphometric analyses

Microscopic images were adjusted using Image J software (NIH) to enhance brightness and contrast for convolutions to emphasize the edges of each mitochondrial particle. After the threshold was modified, individual mitochondria were analyzed for circularity and major/minor axes. Foam factor (FF: circularity value) and aspect ratio (AR: major/minor axis of an ellipse equivalent to the object) were calculated, and indicated as a scatter plot of AR versus FF in a graph for each image. Both parameters have a minimal value of 1 as a small perfect circle and the increase of value indicated as elongated mitochondria. Specifically, the value of AR indicates mitochondrial length, and FF indicates both mitochondrial length and branching.

### *In vitro* microRNA transfection

Transfections of miRNA mimics were performed using siLentFect™ Lipid reagent (Life Science Research). Mature specific miRNA-182 or anti-miRNA-182 and negative control miR (Genolution Pharmaceuticals, Inc., Korea) were used a final concentration of 100 and 20 to 100 nM for miRNA-182 and for anti-miRNA-182, respectively. After 4 hr incubation in a CO_2_ incubator at 37 °C, the medium was changed to 10% FBS α-MEM. For co-delivery of miR-182 and anti-miR-182, miR/lipid and anti-miRNA/lipid complex were prepared separately, but added to culture medium simultaneously.

### Luciferase reporter assay

The predicted selected miRNAs targeting bnip3 was retrieved using publicly available databases (TargetScan and miRWalk). The 3′-UTR of Bnip3 was cloned into the pmirGLO vector (pmiR-GLO Bnip3-3′UTR). For mutant pmirGLO vector (pmiR-GLO scrambled Bnip3-3′UTR), scrambled miR-182 binding sequence (GCUAGUA) was used instead of wild miR-182 binding sequence (UUGCCAA). HeLa was plated at 1 × 10^5^ in 12-well culture plates. After 24 h, the pmirGLO vector containing the Bnip3 binding site for miRNAs was co-transfected with the negative control using Lipofectamine 2000. The Renilla luciferase was used to normalize. Luciferase activity was measured by using Dual Luciferase assay (Promega) according to the manufacturer’s instructions. Each assay was repeated three times. The luciferase activity of the cells only transfected with each vector (pmiR-GLO, pmiR-GLO Bnip3-3′UTR, and pmiR-GLO scrambled Bnip3-3′UTR) served as controls for each vector group with relative value of 1.

### Measurement of ROS

To measure cytosolic ROS production in cardiomyocytes, cytosolic specific staining with cytosolic (CM-H2DCFDA) was used. The cells were plated at a density of 5 × 10^5^ cells/well in 35 mm plates and the cells were harvested with trypsin-EDTA 6 hr after treatments. The growth media from cells was removed and then the cells were re-suspended in pre-warmed PBS at a final working concentration of 10 μM CM-H2DCFDA dye for cytosolic ROS detection. The cells were incubated at 37 °C for 10 min and then returned to pre-warmed growth medium, followed by incubation at 37 °C for 10 min to render the dye responsive to oxidation. After that step, the cells were washed with PBS twice, and were replaced to the warmed medium containing 4% formalin. The cells were incubated at 37 °C for 15 min and then washed two times with PBS. The cells were re-suspended in 300 μl PBS and analyzed by FACSVerseTM flow cytometer (BD biosciences). Data were analyzed using FlowJo Version 7.5.4 software.

### Ischemia-reperfusion injury

Myocardial infarction was produced in 8-week-old Sprague-Dawley male rats (250 g) by surgical occlusion of the left anterior descending coronary artery. Briefly, under general anesthesia with zoletil (20 mg/kg) and zylazine (5 mg/kg), rats were intubated, and positive-pressure ventilation (12 ml/kg) was maintained with room air supplemented with oxygen (70 strokes/min, tidal volume: 8–10 ml/kg) using a Harvard ventilator. The third and fourth ribs were cut to open the chest, and heart was exteriorized through the intercostal space. The heart was exposed through a 2-cm left lateral thoracotomy. The pericardium was incised and a 6-0 silk suture (Johnson & Johnson, Langhome, PA) was placed around the proximal portion of the left coronary artery, beneath the left atrial appendage. Ligature ends were passed through a small length of plastic tube to form a snare. For coronary artery occlusion, the snare was pressed onto the surface of the heart directly above the coronary artery and a hemostat applied to the snare. Ischemia was confirmed by the blanching of the myocardium and dyskinesis of the ischemic region. After 60 min of occlusion, the hemostat was removed and the snare released for reperfusion, with the ligature left loose on the surface of the heart. Restoration of normal rubor indicated successful reperfusion. Wounds were sutured and the thorax was closed under negative pressure. Rats were weaned from mechanical ventilation and returned to cages to recover. For delivery of compound **5** (100 μM, 50 μl per animal), intravenous injection to the femoral vein was used. To deliver the miRNA mimc to heart tissue, local oligonucleotide delivery model was used with F-127 pluronic gel (Sigma) as previously described^45^. MicroRNA-182 mimic (90 μg/animal) was mixed with transfection solution (0.2% transfection reagent in Opti-MEM, 50 μl) and the mixture was preloaded into 400 μl 30% F-127 pluronic gel at 4 °C. After the myocardial infarction, the mixture was applied locally to the infarcted area.

### TUNEL assay

ApopTag Plus Fluorescein *In Situ* Kit was used. For *in vivo* paraffin sections, the tissue sections washed in PBS twice for 5 min each after de-paraffinization. The tissue sections were treated with Proteinase K (20 μg/ml) for 15 min at room temperature. The specimens were washed twice in coplin jar in tap water for 2 min each and quenched in 3% hydrogen peroxide in PBS for 5 min at room temperature. The specimen was rinsed twice with PBS or water for 5 min each time in a coplin jar. Gently, the samples tapped off and equilibration buffer was applied for a least 10 sec at room temperature. And then, the sections were treated with working strength TdT enzyme to incubate in a humidified chamber at 37 °C for 1 hr. The specimen were agitated with stop/wash buffer for 15 sec and incubated for 10 min at room temperature. The sections were washed in 3 changes of PBS for 1 min each wash and Anti-digoxignenin conjugate was applied to the slides for 30 min in incubator under humid condition at room temperature. The specimen washed 4 times with PBS for 2 min per wash at room temperature and then was treated with peroxidase substrate to completely cover them for 3 to 6 min. The specimen was washed in 3 changes of D.W. Counter-staining was performed with 0.5% methyl-green solution for 20 min at room temperature. Dehydration was performed using 100% N-butanol, ethanol and zylene. For quantification, five sections of each group were prepared and five different regions per slide were chosen for observation.

### Left ventricular catheterization for functional analysis of heart

For invasive hemodynamics, left ventricular catheterization was performed at 3 weeks after operation. A Millar Micro-tip 2 F pressure transducer (model SPR-838, Millar Instruments, USA) was introduced into the left ventricle via the right carotid artery under zoletil (20 mg/kg) and xylazine (5 mg/kg) anesthesia. Real time pressure volume loops were recorded by a blinded investigator and all data were analyzed with PVAN 3.5 software (Millar).

### Statistical analysis

Quantitative data were expressed as the means ± S.E.M of at least 3 independent experiments. For statistical analysis, one-way ANOVA with Bonferroni correction was performed using the OriginPro 8 SR4 software (ver. 8.0951, OriginLab Corporation, Northampton, MA, USA). A *p* value of less than 0.05 was considered to be statistically significant.

## Additional Information

**How to cite this article**: Lee, S.-Y. *et al.* Small molecule-mediated up-regulation of a microRNA targeting a key cell death modulator BNIP3 improves cardiac function following ischemic injury. *Sci. Rep.* **6**, 23472; doi: 10.1038/srep23472 (2016).

## Supplementary Material

Supplementary Information
