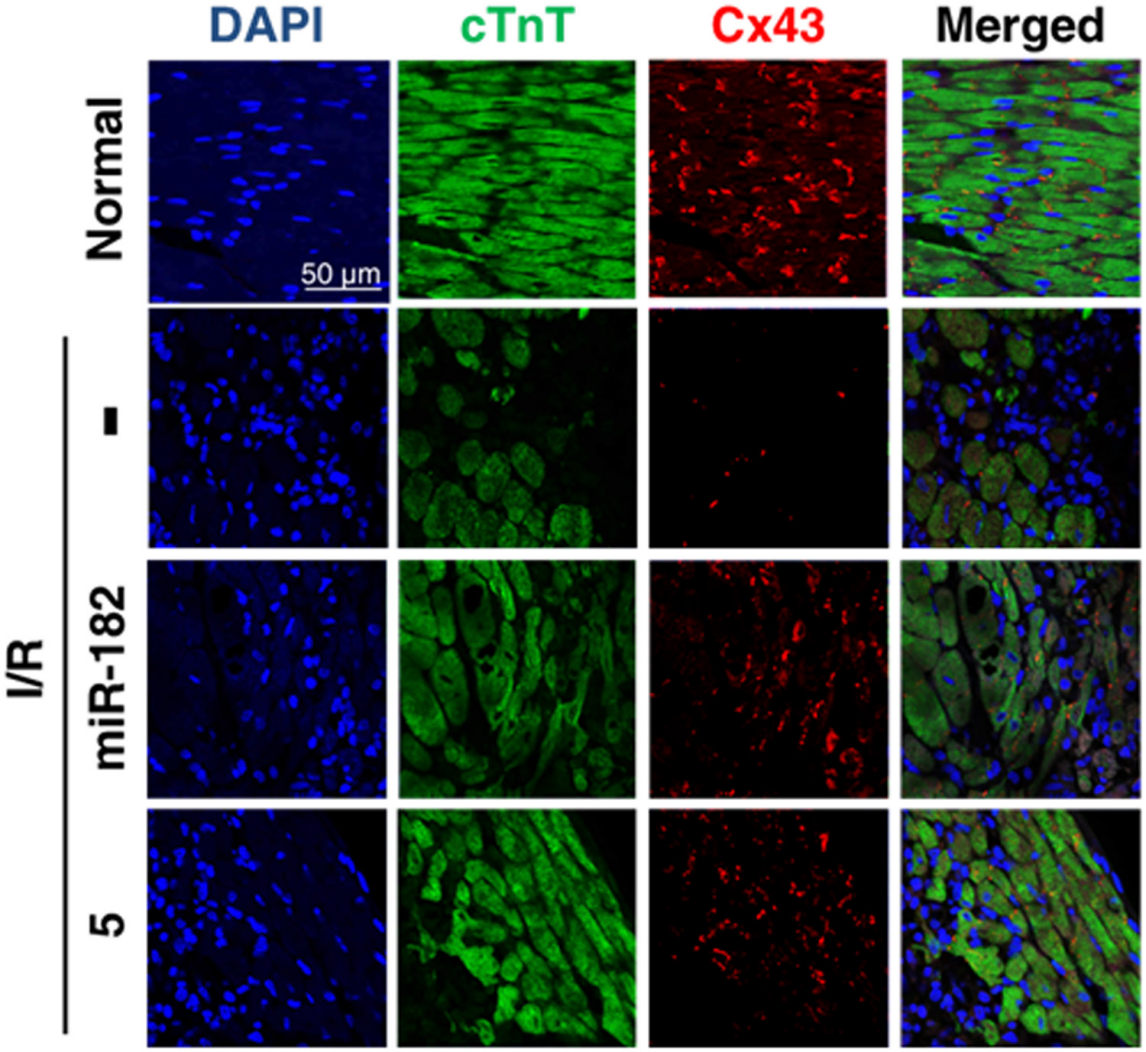# Corrigendum: Small molecule-mediated up-regulation of microRNA targeting a key cell death modulator BNIP3 improves cardiac function following ischemic injury

**DOI:** 10.1038/srep46973

**Published:** 2018-05-17

**Authors:** Se-Yeon Lee, Seahyoung Lee, Eunhyun Choi, Onju Ham, Chang Youn Lee, Jiyun Lee, Hyang-Hee Seo, Min-Ji Cha, Bohyun Mun, Yunmi Lee, Cheesoon Yoon, Ki-Chul Hwang

Scientific Reports 6: Article number: 2347210.1038/srep23472; published online: 03 24 2016; updated: 05 17 2018

The Article contains errors in Figure 4A, and Supplementary Figures 11, 12 and 13. As a result of the misfiling of the data, the images labelled as Compound 5 are of kenpaullone-treated group.

The correct figures 4A, S11, S12 and S13, showing the correct data for Compound 5, appear below as Figures 1, 2, 3 and 4 respectively.

The conclusions of the Article are unaffected by the correction. The authors apologize for the errors and any confusion caused.

## Figures and Tables

**Figure 1:**
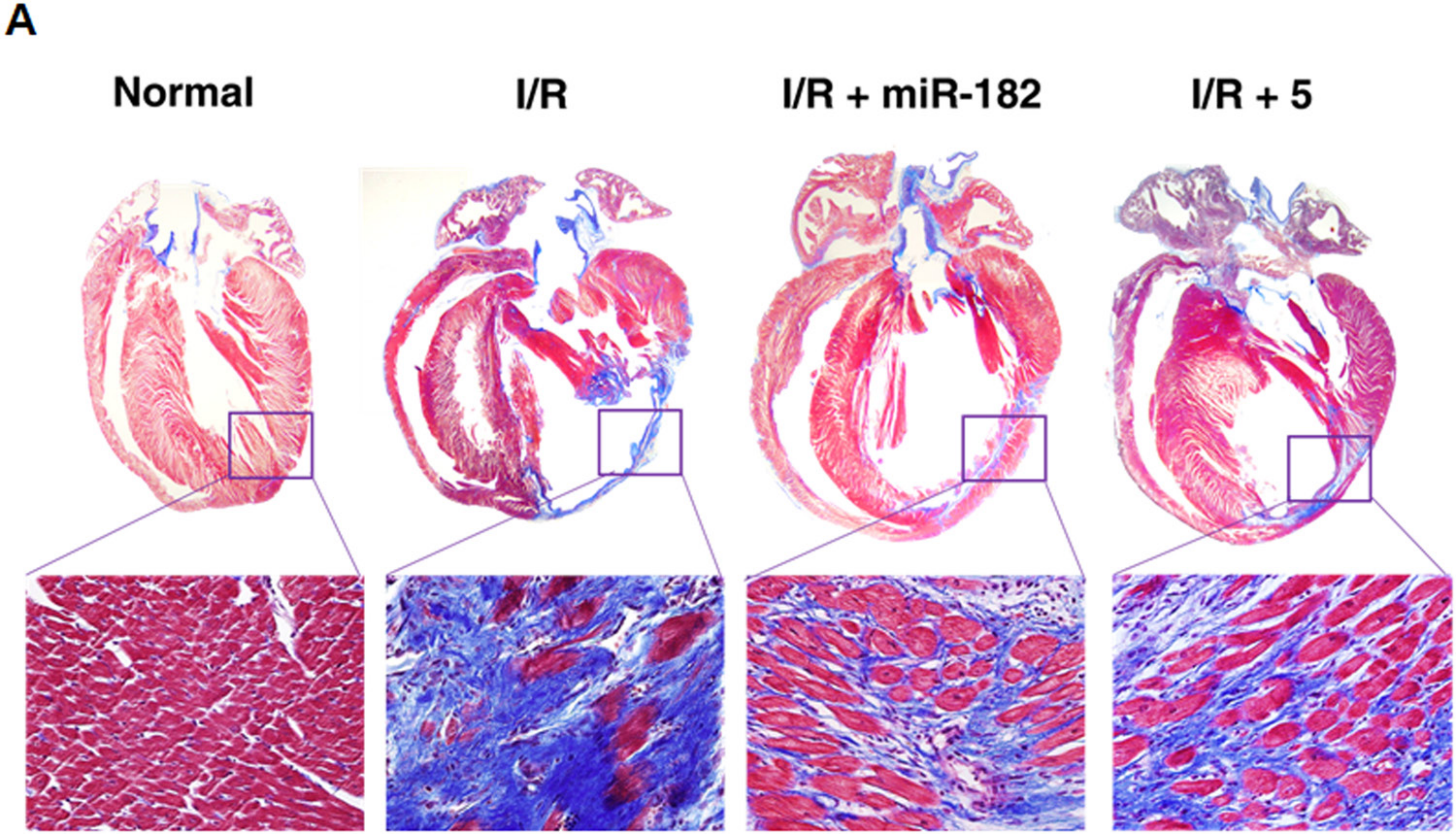


**Figure 2:**
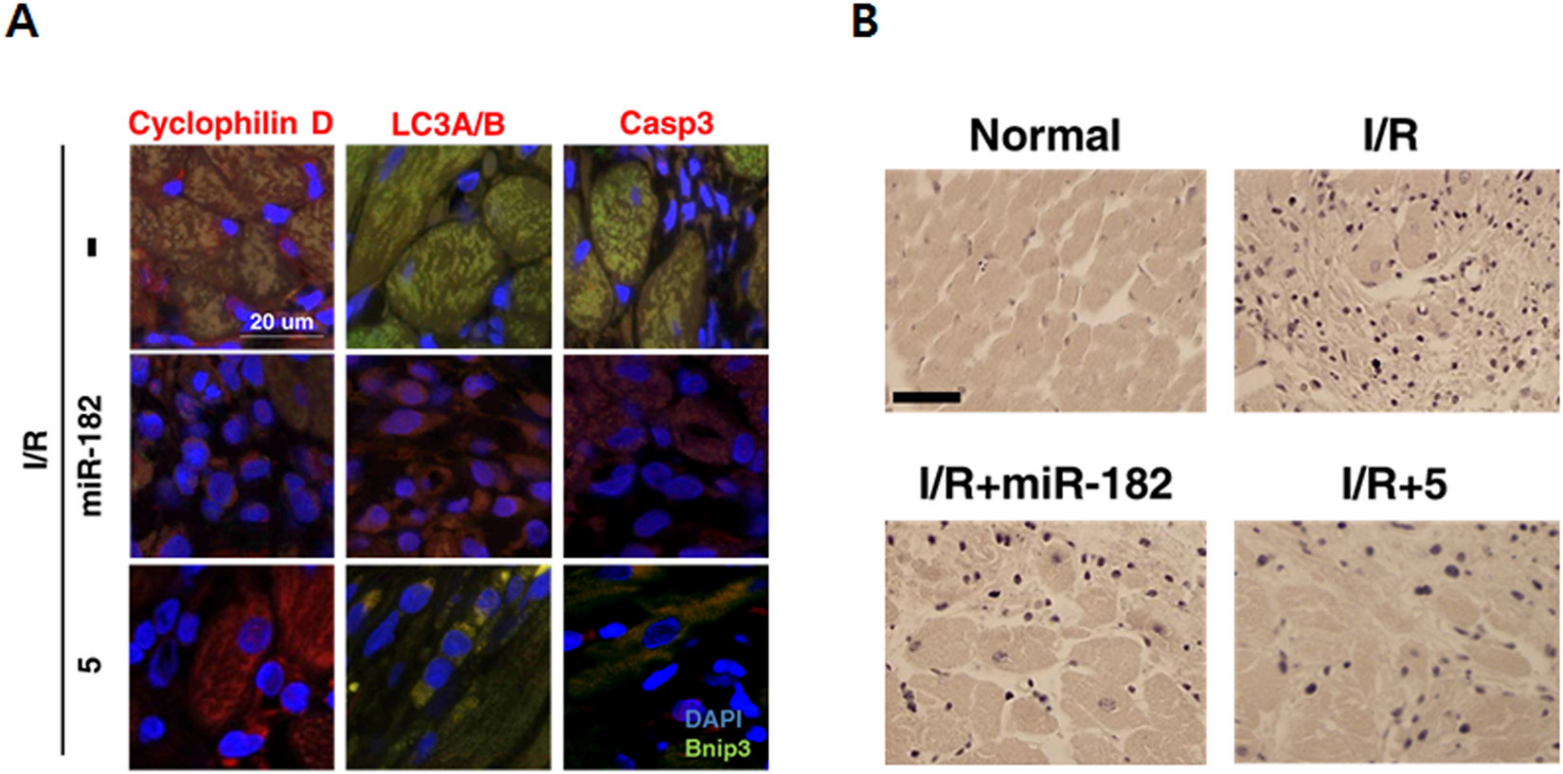


**Figure 3:**
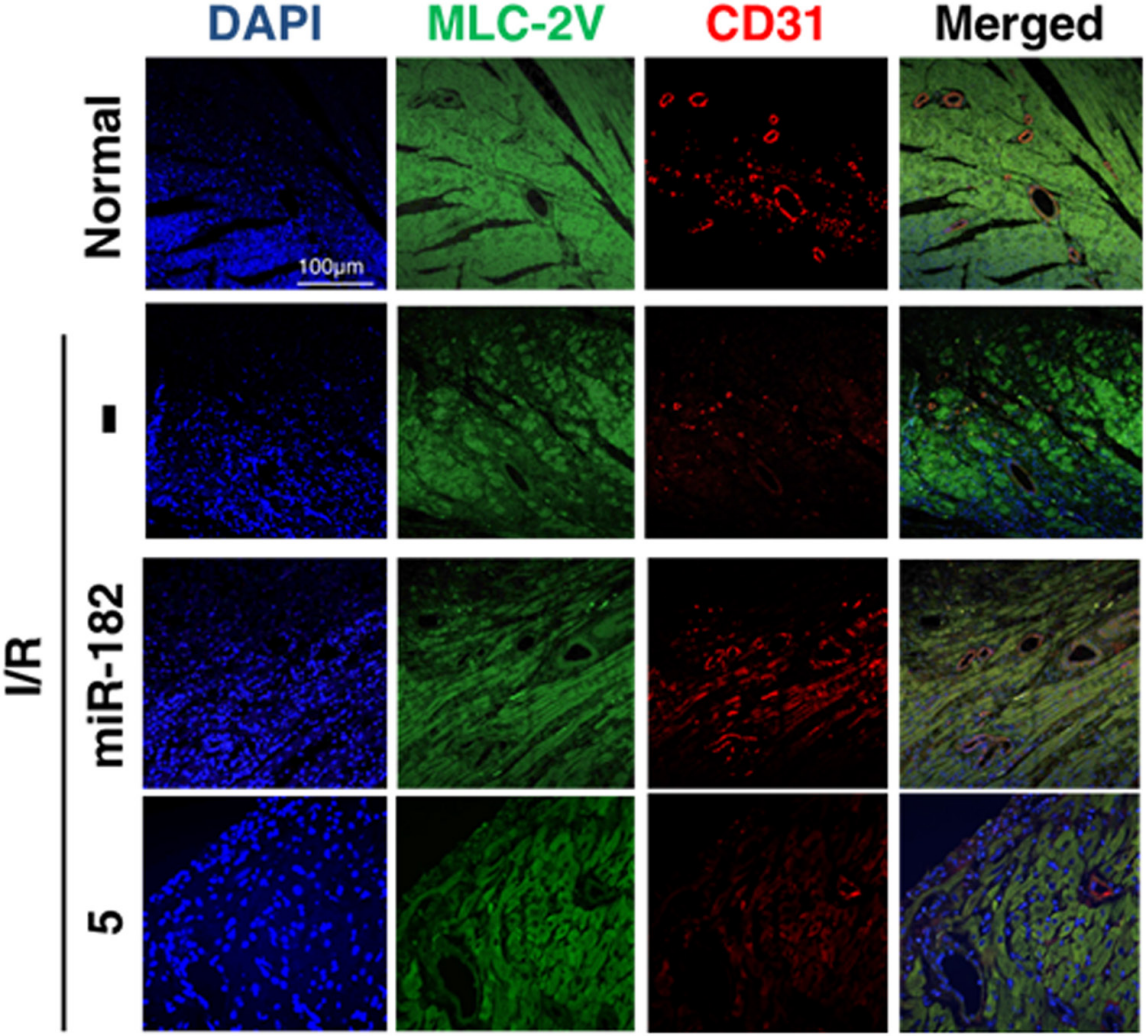


**Figure 4:**