# Supplementary material for: Small molecule-mediated up-regulation of microRNA targeting a key cell death modulator BNIP3 improves cardiac function following ischemic injury
Source: Sci Rep. 2016 Mar 24;6:23472. doi: 10.1038/srep23472 (PMC4806297; doi:10.1038/srep23472)
Supplement: Supplementary Information [file srep23472-s1.pdf]

Supplementary Information

Small molecule-mediated up-regulation of microRNA targeting a key cell death modulator BNIP3 improves cardiac function following ischemic injury

Se-Yeon Lee, Seahyoung Lee, Eunhyun Choi, Onju Ham, Chang Youn Lee, Jiyun Lee, Hyang-Hee Seo, Min-Ji Cha, Bohyun Mun, Yunmi Lee, Cheesoon Yoon, and Ki-Chul Hwang*

Supplementary Figures

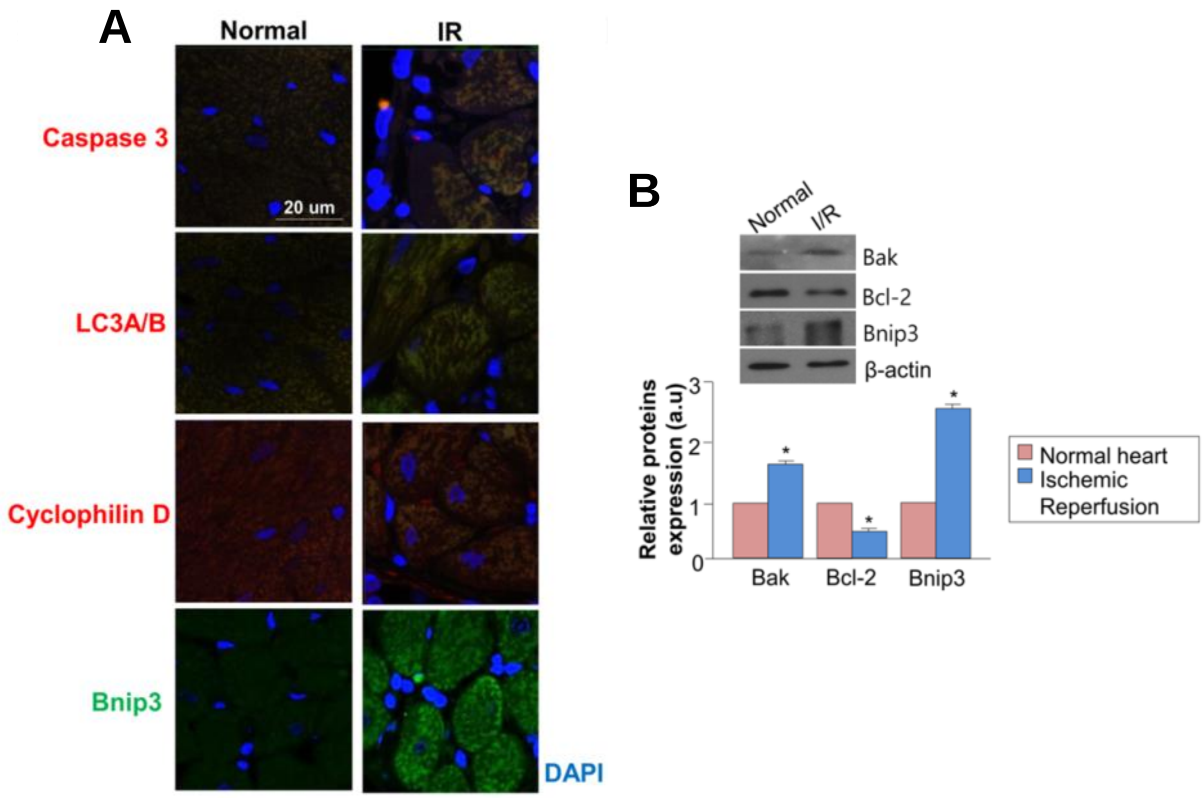

Supplementary Figure 1. Ischemia-reperfusion injury induced different types of cell death in heart. (A) Following IR-injury (7d), markers of apoptosis (Caspase3), autophagy (LC3A/B), and necrosis (cyclophilinD) were detected by immunohistochemistry along with the expression of BNIIP3. (B) Expressions of apoptosis-related factors (Bak and Bcl-2), as well as BNIP3, in IR-injured heart were detected by western blot. *$p<0.05$ compared to normal control.

A

MicroRNA	DIANAmT	miRanda	miRDB	miRWalk	PICTAR4	PICTAR5	PITA	RNA22	Targetscan	microRNA.org	SUM
rno-miR-411	0	1	1	1	0	0	1	0	1	1	**6**
rno-miR-336	0	1	1	1	0	0	1	0	1	0	**5**
rno-miR-142 -5p	0	1	1	1	0	0	1	0	1	0	**5**
rno-miR-137 -3p	0	1	0	1	0	0	1	0	1	1	**5**
rno-miR-873	0	1	0	1	0	0	1	0	1	1	**5**
rno-miR-182	0	1	1	1	0	0	1	0	0	1	**5**
rno-miR-224 -5p	0	1	0	0	0	0	1	0	1	1	**4**
rno-miR-291-3p	0	1	0	1	0	0	1	0	0	1	**4**
rno-miR-300-3p	0	1	0	1	0	0	1	0	0		3
rno-miR-301b	0	1	0	0	0	0	1	0	1		3
rno-miR-495	0	1	0	1	0	0	1	0	0		3
rno-miR-329	0	1	0	1	0	0	1	0	0		3
rno-miR-93	0	1	0	1	0	0	1	0	0		3
rno-miR-451	0	1	0	1	0	0	1	0	0		3
rno-miR-412	0	1	0	1	0	0	1	0	0		3

B

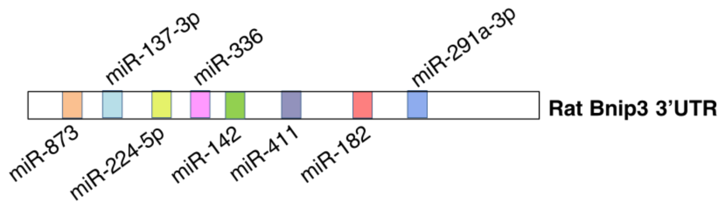

Position (of bnip3 3'UTR)	miRNA	Sequence (3' -> 5')
Position 685-691	miR-411	GCAUGCGAUAUGCCAGAUGAU
Position 283-289	miR-137-3p	GAUGCGCAUAAGAAUUCGUUAUU
Position 127-134	miR-873	UCCUCUGAGUGUUCAAGGACG
Position 723-729	miR-182	GGAAAUGAAAAAAAUCUUUGCCAAA
Position 350-356	miR-224-5p	UUUGCCUUGGUGAUCACUGAAC
Position 776-772	miR-291-3p	AAUUAAUCAGUAUCCAGCACUUG
Position 435-441	miR-336	UCUGAUCUAUACC-UUCCCACU
Position 465-471	miR-142	UCAUCACGAAAGAUGAAAUAC

Supplementary Figure 2. Screening of candidate miRNAs targeting BNIP3. (A) Using miRNA target prediction database (TargetScan and miRWalk), 8 candidate miRNAs that are predicted by more than 4 databases were selected for validation. (B) Relative positions of binding sites for selected miRNAs in the 3'UTR of BNIP3.

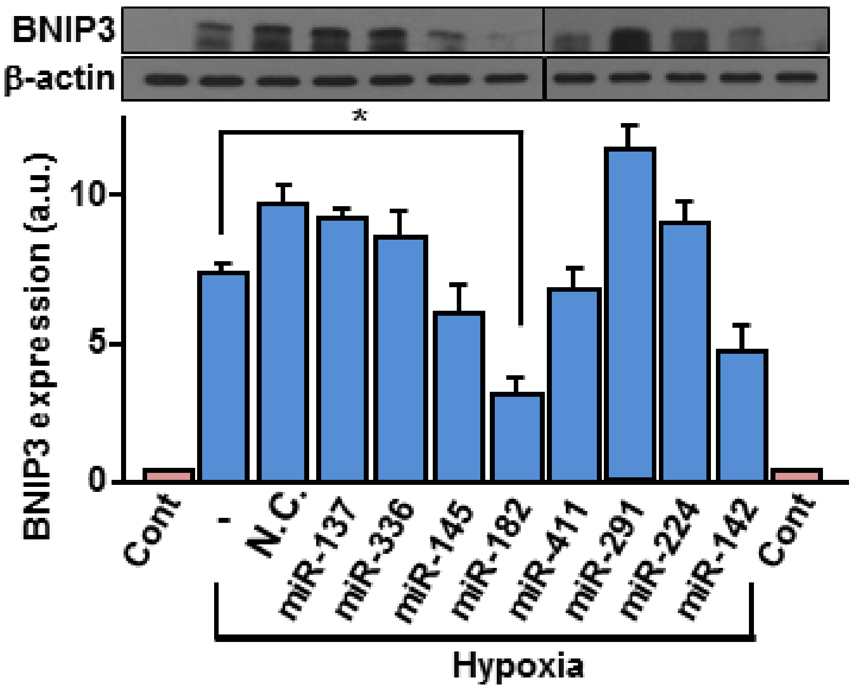

Supplementary Figure 3. Screening of BNIP3-targeting miRNAs. Carrdiomyocytes were transfected with candidate miRNAs (100nM, each) for 24 hours prior to 24 hours of hypoxia treatment. BNIP3 expression was detected by Western blot. *p<0.05.

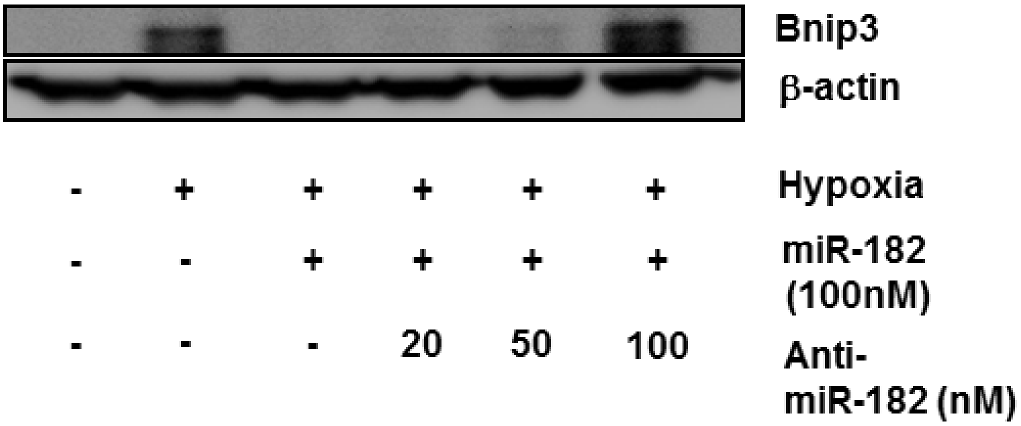

Supplementary Figure 4. Effect of anti-miR-182 on miR-182-induced downregulation of BNIP3 under hypoxia. Cardiomyocytes were transfected with miR-182 and increasing concentrations of anti-miR-182 for 24 hours prior to 24 hours of hypoxia treatment. BNIP3 expression was detected by Western blot.

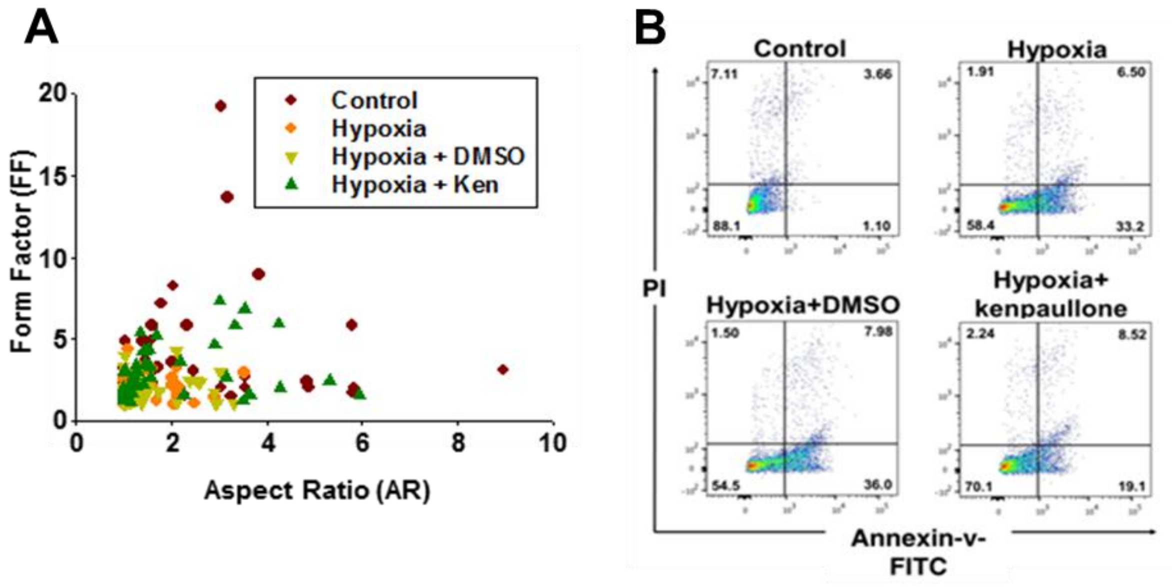

Supplementary Figure 5. Kenpaullone prevents hypoxia induced mitochondrial fission and apoptosis. (A) Quantification of mitochondrial fission. (B) Flow cytometry for detecting apoptosis. PI: propidium iodide.

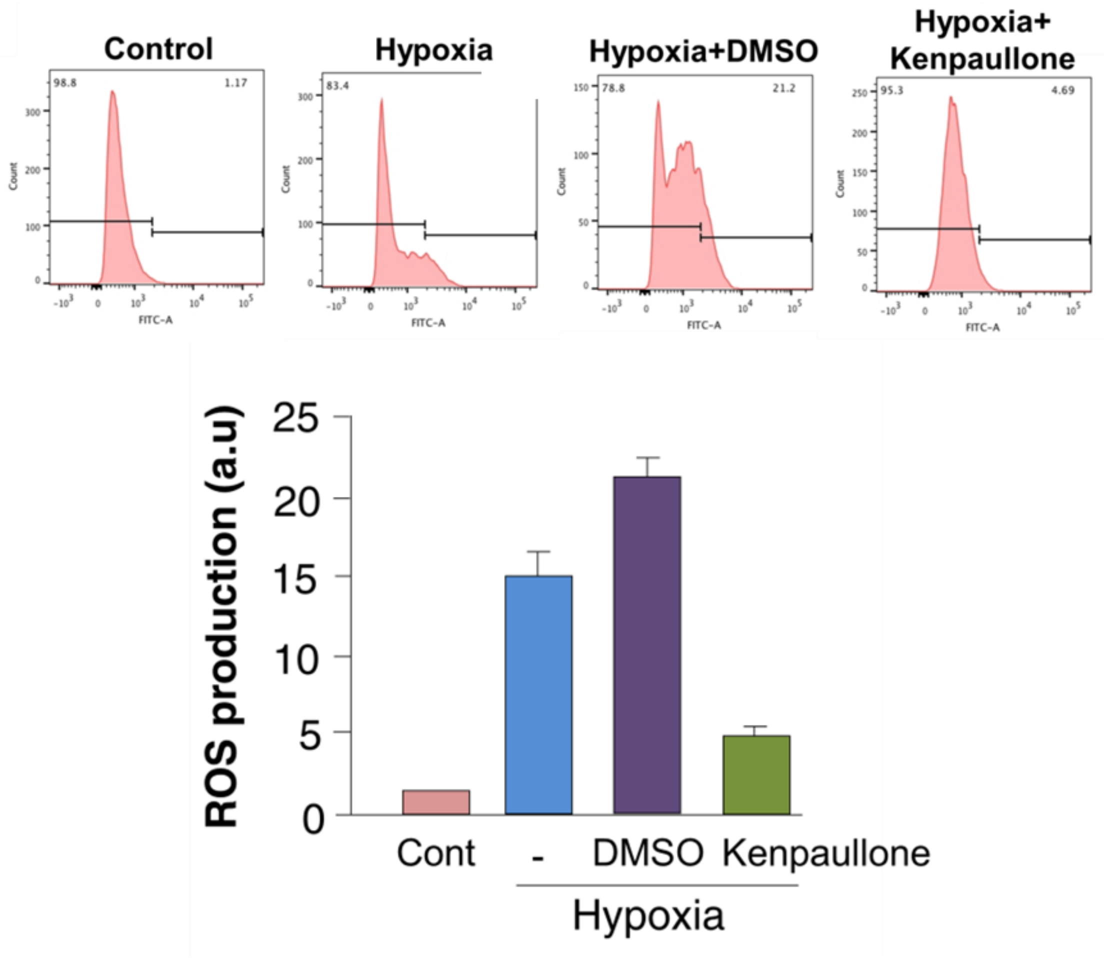

Supplementary Figure 6. Kenpaullone attenuates hypoxia-induced reactive oxygen species (ROS) production. Effect of kenpaullone (10μM) on hypoxia-induced ROS production.

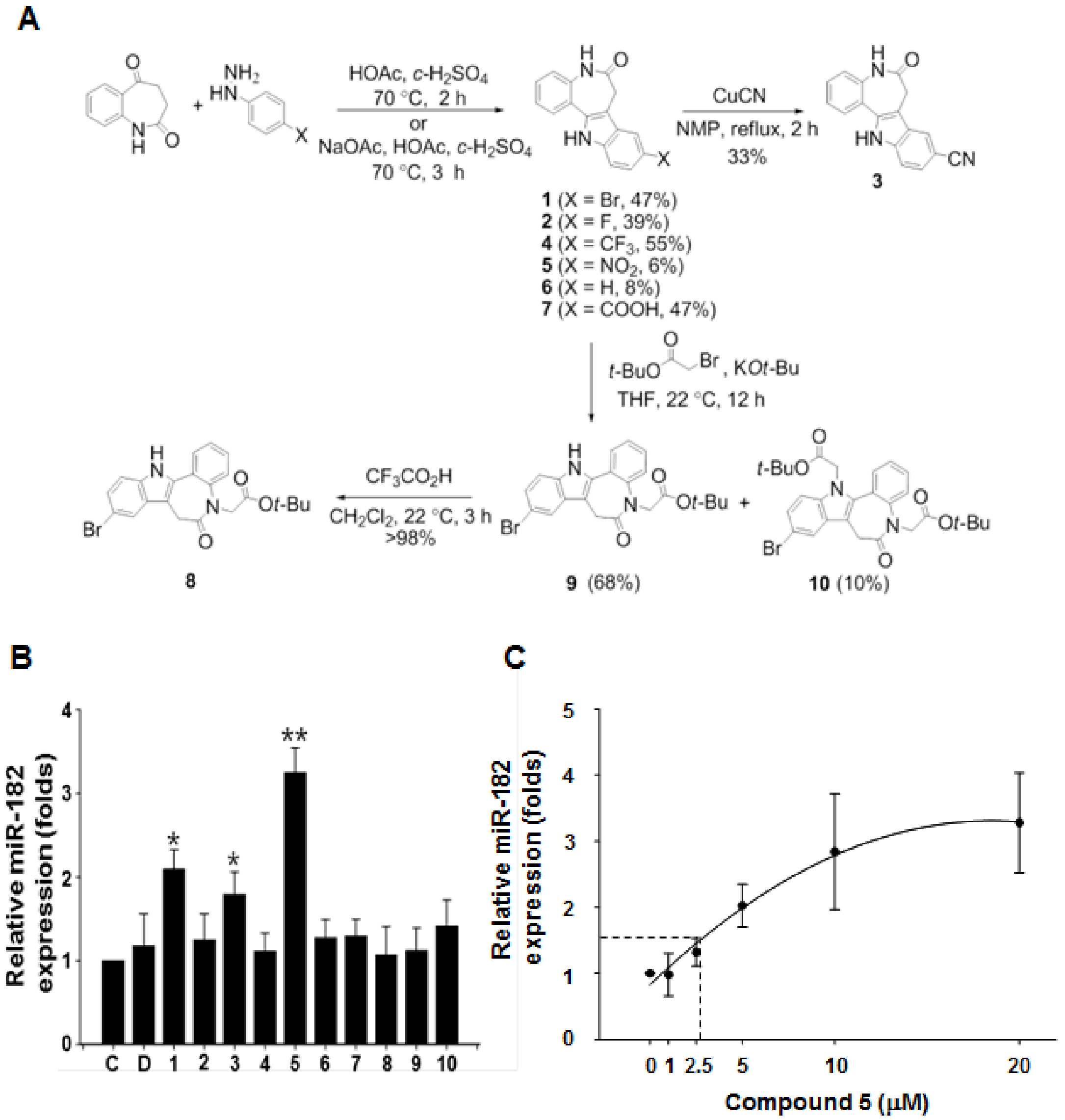

Supplementary Figure 7. Compound **5**, a derivative of kenpaullone, induces miR-182 expression. (A) Schematics of general synthesis of kenpaullone derivatives. (B) Cardiomyocytes were treated with 10 different compounds (kenpaullone: **1** and 9 derivatives, 10 µM each) for 24 hours, and the expression of miR-182 was evaluated by real-time PCR. C: control; D: DMSO; see Scheme 1 for compound numbering. *p < 0.05 compared with control. **p < 0.05 compared with kenpaullone (**1**)-treated group. (C) Dose-response curve for compound **5**-induced miR-182 expression. The results indicated that an EC_{50} value of approximately 2.5 µM.

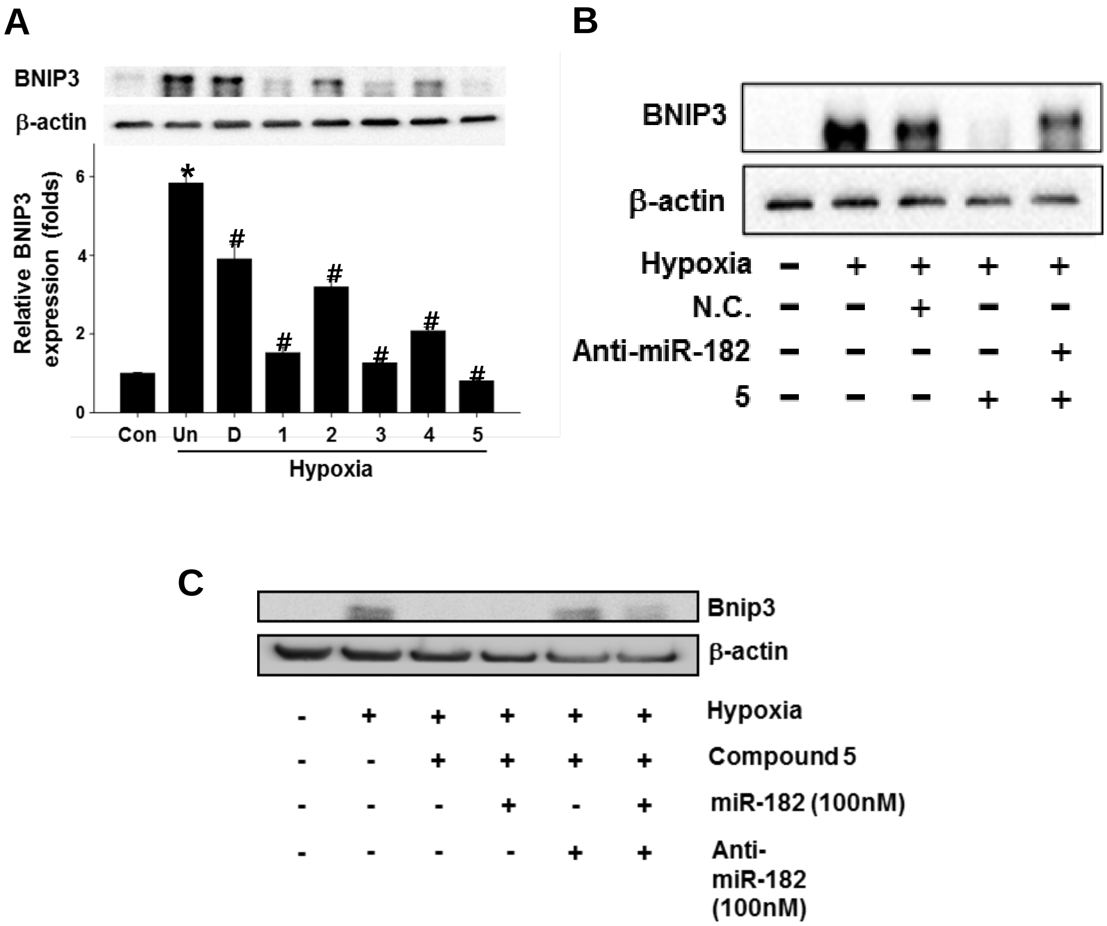

Supplementary Figure 8. Effect of kenpaullone derivatives on BNIP3 expression under hypoxia. (A) Cells were pre-treated with 4 different kenpaullone (**1**) derivatives (**2**-**5**) prior to hypoxia treatment. The expression of BNIP3 was measured by Western blot. Un: untreated; D: DMSO. *p<0(B) Cells were pre-treated with compound 5 and/or anti-miR-182 (50nM) prior to hypoxia treatment. The expression of BNIP3 was measured by Western blot. N.C: negative control. (C) To examine the effect of anti-miR-182 on 5-mediated down-regulation of BNIP3, miR-182 and anti-miR-182 were co-delivered.

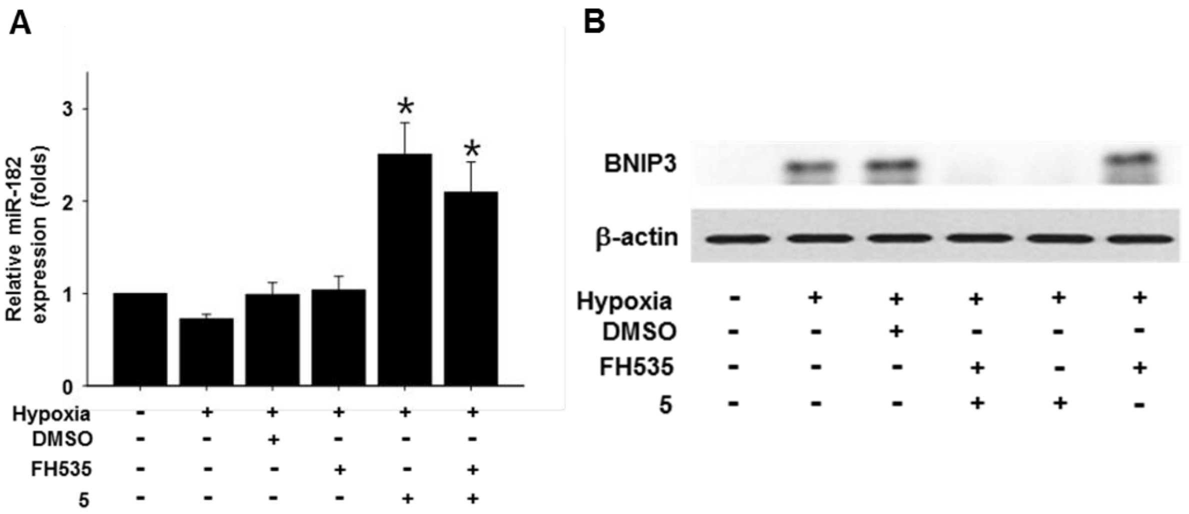

Supplementary Figure 9. Effect of β-catenin inhibitor on 5-induced miR-182 and BNIP3 expression. (A) Cells were pre-treated with or without β-catenin inhibitor (FH535, 100 μM) prior to compound 5 treatment. Relative miR-182 expression was evaluated by real-time PCR. (B) Cells were pre-treated with or without β-catenin inhibitor (FH535, 100 μM) prior to compound **5** treatment. BNIP3 expression was evaluated by Western blot.

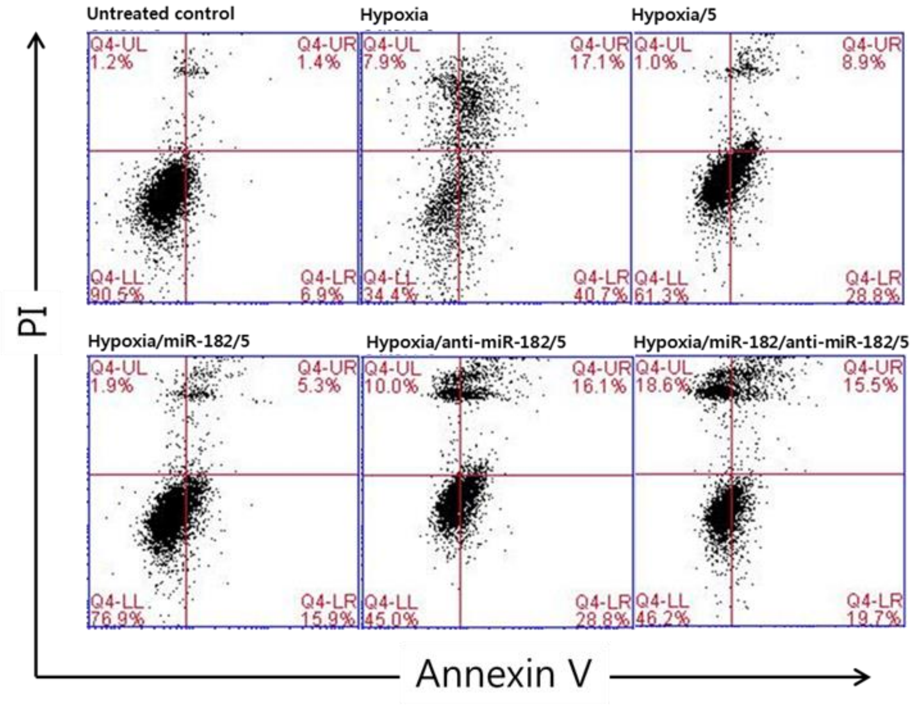

Supplementary Figure 10. The effect of anti-miR-182 on the compound 5 or miR-182-mediated anti-apoptotic effect. Cellular apoptosis of cardiomyocytes exposed to 24 hours of hypoxia with treatment of 5, miR-182 (100nM), anti-miR-182 (100nM) or their combinations as indicated was evaluated by flow cytometry using Annexin V/PI staining.

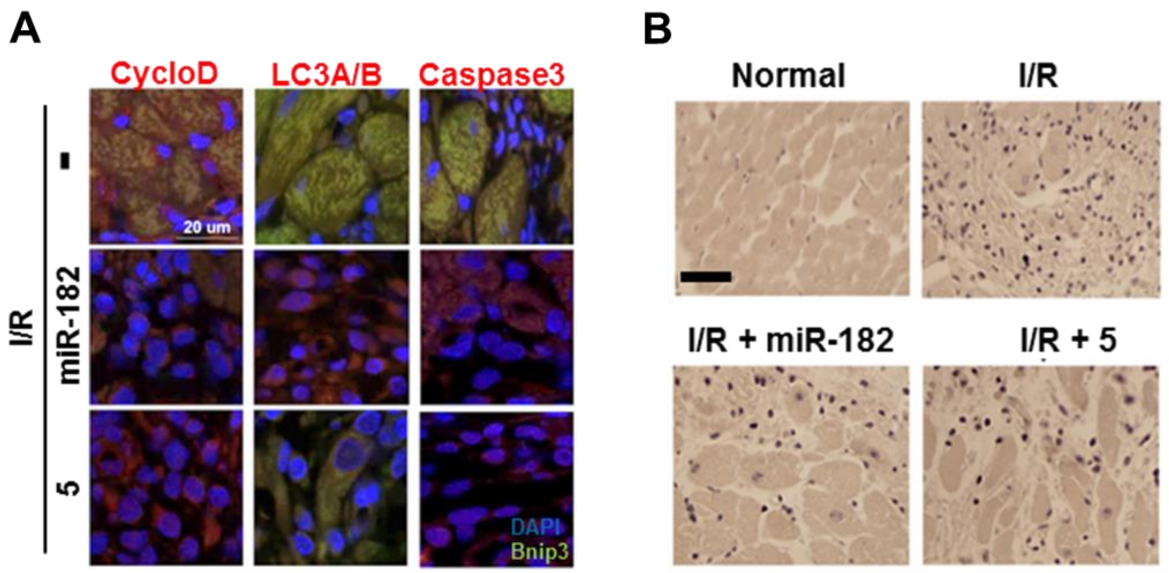

Supplementary Figure 11. Compound 5 prevents I/R injury-induced cardiac cell death *in vivo*. (A) Three weeks after the I/R-injury, markers of different cell death mechanisms were detected in the heart treated with either miR-182 (90 μg per animal) or **5** (100 μM, 50μl per animal) by immunohistochemistry. CycloD (cyclophilin D, necrosis), LC3A/B (autophagy), Caspase3 (apoptosis). (B) Apoptosis in the heart was evaluated by TUNEL staining. Scale bar = 100 μm.

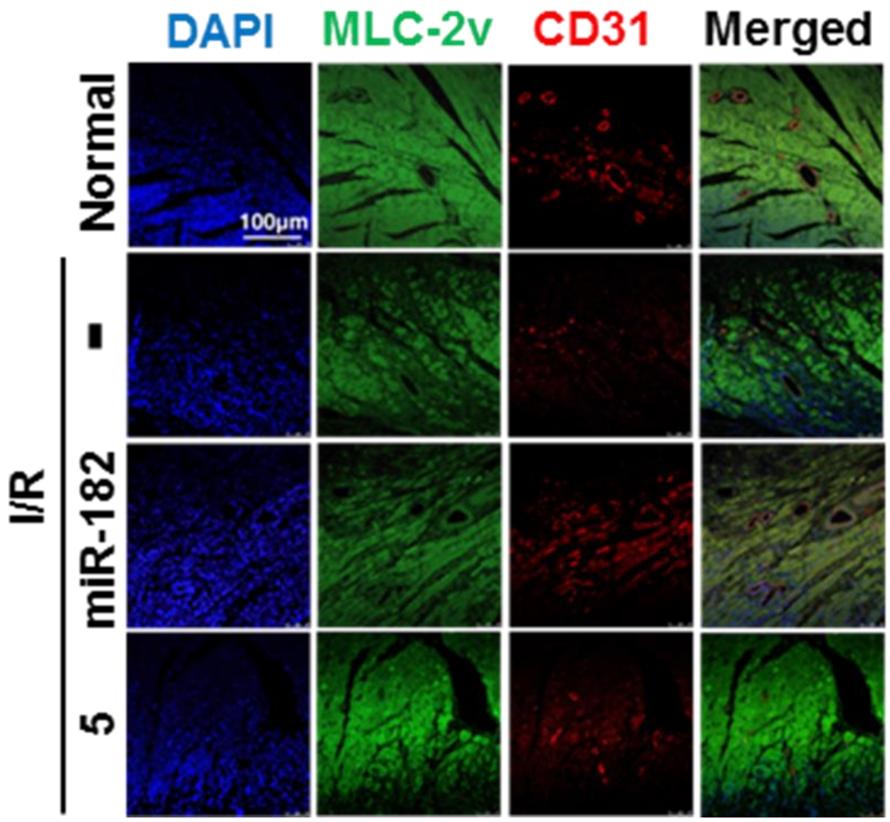

Supplementary Figure 12. Effect of 5 and miR-182 on the expressions of MLC-2v and CD31 in IR-injured heart. Following IR-injury (7d), the expressions of myosin light chain 2v (MLC-2v) and CD31 were examined by immunohistochemistry. Nuclei were counterstained with DAPI.

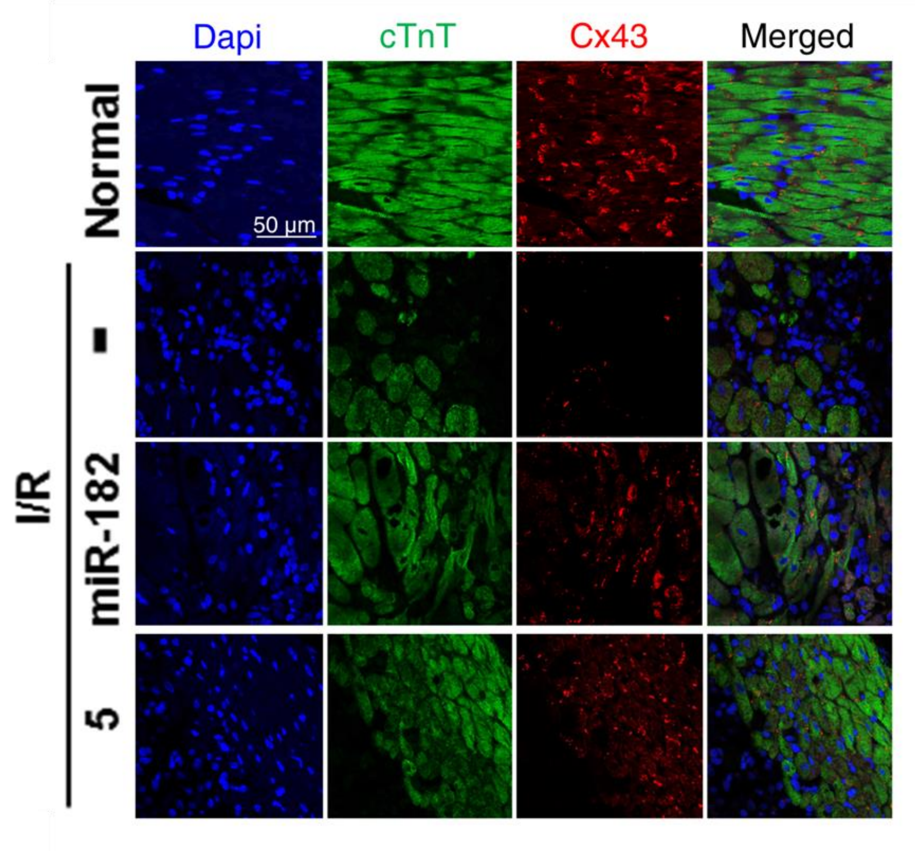

Supplementary Figure 13. Effect of 5 and miR-182 on the expressions of cTnT and Cx43 in IR-injured heart. Following IR-injury (7d), expressions of cardiac troponin T (cTnT) and connexin 43 (Cx43) were examined by immunohistochemistry. Nuclei were counterstained with DAPI.

Supporting Information (chemical synthesis)

General. Infrared (IR) spectra were recorded on a MB104 (ABB Bomen, InC) spectrophotometer, νmax in cm^{-1}. Bands are characterized as broad (br), strong (s), medium (m), and weak (w). ^{1}H NMR spectra were recorded on a JEOL JNM-AL400 (400 MHz) spectrometer. Chemical shifts are reported in ppm from tetramethylsilane, with the solvent resonance as the internal standard ($CDCl_3$: δ 7.27 ppm). Data are reported as follows: chemical shift, multiplicity (s = singlet, d = doublet, t = triplet, q = quartet, m = multiplet), coupling constants (Hz), and integration. ^{13}C NMR spectra were recorded on a JEOL JNM-AL400 (100 MHz) spectrometer with complete proton decoupling. Chemical shifts are reported in ppm from tetramethylsilane with the solvent resonance as the internal standard ($CDCl_3$: δ 77.26 ppm). High-resolution (HR)-electrospray ionization (ESI) mass spectra were obtained on a SYNAPT G2 (Waters, U.K.) ESI-TOF mass spectrometer.

Unless otherwise noted, all reactions were carried out with distilled solvents under an atmosphere of dry N_2 in oven-dried (130 °C) glassware. Dichloromethane was distilled over calcium hydride and tetrahydrofuran was purified by distillation from sodium benzophenone ketyl immediately prior to use unless otherwise specified. All work-up and purification procedures were carried out with reagent grade solvents in air.

■ **Representative experimental procedure A:**

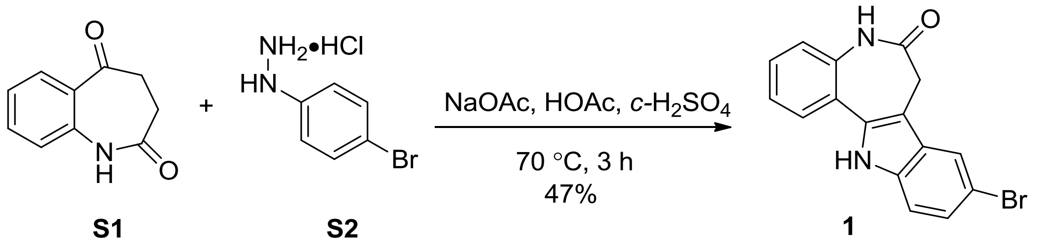

9-Bromo-7,12-dihydrobenzo[2,3]azepino[4,5-b]indol-6(5H)-one (1). A solution of 3,4-dihydro-1H-benzo[b]azepine-2,5-dione (**S1**) [1] (87.0 mg, 0.497 mmol), sodium acetate (61.0 mg, 0.745 mmol) and 4-bromophenylhydrazine chloride (**S2**) (167 mg, 0.745 mmol) in glacial acetic acid (3.3 mL) was allowed to heat to 70 °C and stir for 1 h. After that time, concentrated H_2SO_4 (33 μL) was added to the reaction solution and stirring was continued at 70 °C for 1 h. Then, additional portion of concentrated H_2SO_4 (33 μL) was added. After stirring for a further 1 hour, the reaction

mixture was allowed to cool to room temperature and poured into a 5% aqueous solution of sodium acetate (7 mL). A precipitate was generated, which was filtered off, washed with water, and purified by silica gel column chromatography (hexanes:EtOAc=1:1) to obtain the desired product **1** (77.0 mg, 0.235 mmol, 47% yield) as a brown solid. (This compound has been previously reported and spectra data match those described [2]). **^{1}H NMR** (DMSO-d_6, 400 MHz): δ 11.81 (s, 1H), 10.12 (s, 1H), 7.91 (brs, 1H), 7.74 (d, J = 7.3 Hz, 1H), 7.41-7.35 (m, 2H), 7.30-7.25 (m, 3H), 3.51 (s, 2H); **^{13}C NMR** (DMSO-d_6, 100 MHz): δ 171.6, 136.2, 135.8, 134.1, 128.6, 128.4, 127.1, 124.6, 123.8, 122.5, 122.4, 120.5, 113.5, 111.8, 107.3, 31.4.

■ **Representative experimental procedure B:**

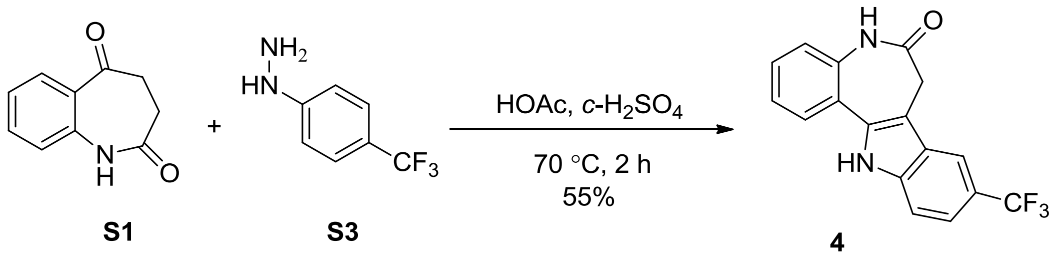

9-(Trifluoromethyl)-7,12-dihydrobenzo[2,3]azepino[4,5-b]indol-6(5H)-one (4). To a solution of 3,4-dihydro-1H-benzo[b]azepine-2,5-dione (**S1**) (50.0 mg, 0.285 mmol) in glacial acetic acid (0.5 mL) was added a solution of 4-(trifluoromethyl)phenylhydrazine (**S3**) (75.4 mg, 0.428 mmol) in glacial acetic acid (2 mL) dropwise with stirring. The reaction mixture was allowed to heat to 70 °C and stir for 1 h. After that time, the solution was allowed to cool to room temperature and concentrated H_2SO_4 (20 μL) was added. After stirring at 70 °C for a further 1 hour, the resulting solution was allowed to cool to room temperature and poured into a 5% aqueous solution of sodium acetate (4 mL). A precipitate was formed, which was filtered off, washed with water, and purified by silica gel column chromatography (hexanes:EtOAc = 1:1), affording the desired product **4** (47.3 mg, 0.156 mmol, 55% yield) as a yellow solid. (This compound has been previously reported and spectra data match those described [2]). **^{1}H NMR** (DMSO-d_6, 400 MHz): δ 12.10 (s, 1H), 10.16 (s, 1H), 8.12 (s, 1H), 7.76 (d, J = 7.8 Hz, 1H), 7.62 (d, J = 8.3 Hz, 1H), 7.47-7.40 (m, 2H), 7.32-7.26 (m, 2H), 3.59 (s, 2H); **^{13}C NMR** (DMSO-d_6, 100 MHz): δ 171.9, 139.1, 136.0, 135.0, 129.0, 127.3, 126.1, 124.1, 122.6, 122.5, 120.3 (q, J = 30.6 Hz), 118.6 (q, J = 3.3 Hz), 116.1 (q, J = 4.1 Hz), 112.4, 108.7, 31.4.

9-Fluoro-7,12-dihydrobenzo[2,3]azepino[4,5-b]indol-6(5H)-one (2). (**2** was prepared according to procedure **A** with 39% yield. This compound has been previously reported and spectra data match those described [2]). **^{1}H NMR** (DMSO-d_6, 400 MHz): δ 11.70 (s, 1H), 10.11 (s, 1H), 7.73 (d, 7.8 Hz, 1H), 7.48 (dd, *J* = 9.7, 2.4 Hz, 1H), 7.44-7.35 (m, 2H), 7.31-7.22 (m, 2H), 7.00 (dt, *J* = 9.0, 9.0. 1.9 Hz, 1H), 3.49 (s, 2H); **^{13}C NMR** (DMSO-d_6, 100 MHz): δ 171.9, 157.3 (d, *J* = 232.5 Hz), 135.7, 134.6, 134.2, 128.5, 127. 1, 124.0, 122.8, 122.5, 112.6 (d, *J* = 10.0 Hz), 110.4 (d, *J* = 25.7 Hz), 108.0, 107.9, 103.0 (d, *J* = 24 Hz), 31.6.

9-Nitro-7,12-dihydrobenzo[2,3]azepino[4,5-b]indol-6(5H)-one (5) (**5** was prepared according to procedure **A** with 6% yield. This compound has been previously reported and spectra data match those described [2]). **^{1}H NMR** (DMSO-d_6, 400 MHz): δ 12.49 (s, 1H), 10.22 (s, 1H), 8.74 (d, *J* = 2.2 Hz, 1H), 8.08 (dd, *J* = 9.0, 2.2 Hz, 1H), 7.78 (d, *J* = 7.8 Hz, 1H), 7.60 (d, *J* = 9.0 Hz, 1H), 7.45 (dd, *J* = 7.7, 7.7 Hz, 1H), 7.34-7.28 (m, 2H), 3.65 (s, 2H); **^{13}C NMR** (DMSO-d_6, 100 MHz): δ 171.5, 141.0, 140.5, 136.3, 136.1, 129.2, 127.2, 126.0, 123.9, 122.5 121.8, 117.5, 115.5, 112.0, 109.8, 31.3.

7,12-Dihydrobenzo[2,3]azepino[4,5-b]indol-6(5H)-one (6) (**6** was prepared according to procedure **B** with 8% yield. This compound has been previously reported and spectra data match those described [2]). **^{1}H NMR** (DMSO-d_6, 400 MHz): δ 11.58 (s, 1H), 10.09 (s, 1H), 7.74 (d, *J* = 7.6 Hz, 1H), 7.66 (d, *J* = 7.8 Hz, 1H), 7.44 (d, *J* = 8.1 Hz, 1H), 7.37 (dd, *J* = 7.6, 7.6 Hz, 1H), 7.30-7.24 (m, 2H), 7.17 (dd, *J* = 7.6, 7.6 Hz, 1H), 7.07 (dd, *J* = 7.5, 7.5 Hz, 1H), 3.50 (s, 2H); **^{13}C NMR** (DMSO-d_6, 100 MHz): δ 171.8, 137.6, 135.5, 132.6, 128.1, 127.0, 126.7, 123.8, 123.0, 122.4, 122.2, 119.2, 118.1, 111.6, 107.7, 31.6.

6-Oxo-5,6,7,12-tetrahydrobenzo[2,3]azepino[4,5-b]indole-9-carboxylic acid (7) (**7** was prepared according to procedure **B** with 47% yield. This compound has been previously reported and spectra data match those described [2]). **^{1}H NMR** (DMSO-d_6, 400 MHz): δ 12.56 (br s, 1H), 11.99 (s, 1H), 10.16 (s, 1H), 8.32 (s, 1H), 7.80 (d, *J* = 8.5 Hz, 1H), 7.76 (d, *J* = 7.8 Hz, 1H), 7.50 (d, *J* = 8.5 Hz, 1H), 7.41 (dd, *J* = 7.3, 7.3 Hz, 1H), 7.31 (d, *J* = 7.5 Hz, 1H), 7.27 (d, *J* = 7.8 Hz, 1H), 3.55 (s, 2H); **^{13}C NMR** (DMSO-d_6, 100 MHz): δ 171.5, 168.4, 139.9, 135.8, 134.2, 128.6, 127.1, 126.2, 123.8, 123.4,

122.4, 121.9, 120.6, 111.3, 108.7, 31.5.

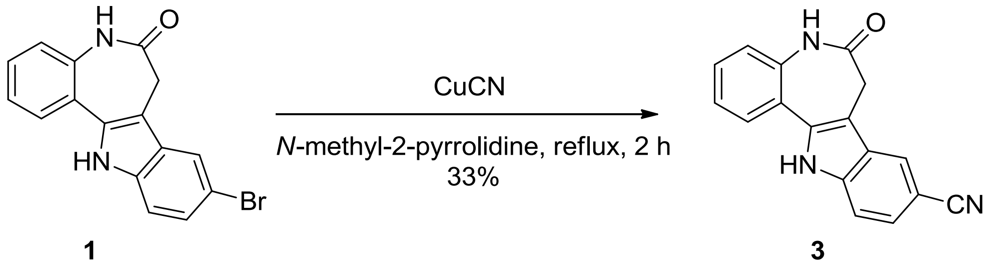

6-Oxo-5,6,7,12-tetrahydrobenzo[2,3]azepino[4,5-b]indole-9-carbonitrile (3). A solution of **1** (18.0 mg, 0.0550 mmol) and copper(I) cyanide (10.0 mg, 0.110 mmol) in *N*-methyl-2-pyrrolidine (0.6 mL) was allowed to reflux for 2 h under N_2 gas. The reaction mixture was allowed to cool to room temperature and poured into water (0.6 mL). A precipitate was formed, which was filtered off, washed with water, and purified by silica gel column chromatography (hexanes:EtOAc = 1:1), affording the desired product **3** (5.00 mg, 0.0183 mmol, 33% yield) as a brown solid. (This compound has been previously reported and spectra data match those described [2]). **^{1}H NMR** (DMSO-d_6, 400 MHz): δ 12.21 (s, 1H), 10.18 (s, 1H), 8.33 (s, 1H), 7.76 (d, *J* = 7.8 Hz, 1H), 7.58 (d, *J* = 8.3 Hz, 1H), 7.51 (d, *J* = 8.6 Hz, 1H), 7.43 (dd, *J* = 7.8, 7.8 Hz, 1H), 7.32 (d, *J* = 7.3 Hz, 1H), 7.28 (d, *J* = 8.3 Hz, 1H), 3.59 (s, 2H); **^{13}C NMR** (DMSO-d_6, 100 MHz): δ 171.6, 139.1, 135.0, 135.1, 129.0, 127.2, 126.5, 124.8, 124.1, 123.9, 122.5, 122.0, 120.7, 112.7, 108.3, 101.3, 31.3.

■ Experimental procedures for the synthesis of compound 8-10:

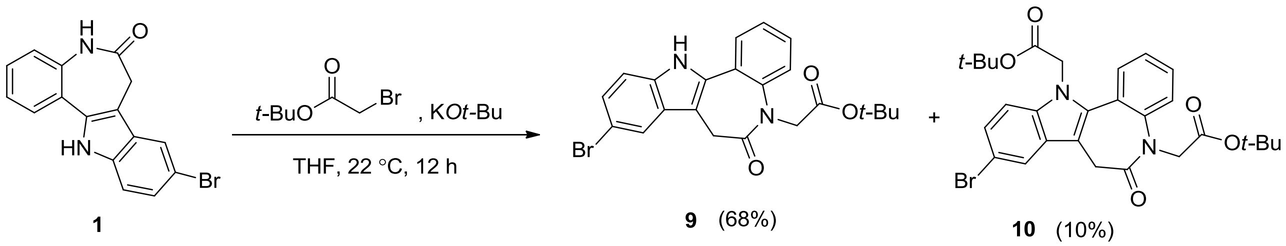

A solution of KO*t*-Bu (1 M in THF, 340 μL) was added to a solution of **1** (100 mg, 0.306 mmol) in THF (5 mL) under N_2 gas. After stirring at room temperature for 1 h, *tert*-butylbromoacetate (50.0 μL 0.336 mmol) was added to the reaction solution, which was allowed to stir for 11 h. After that

time, the resulting solution was quenched with water (5 mL) and washed with CH_2Cl_2 (5 mL x 3). The combined organic layers were dried over $MgSO_4$, filtered, and concentrated *in vacuo*. The crude product was purified by silica gel column chromatography (hexanes:EtOAc = 3:1) to afford the desired product **9** (92.5 mg, 0.210 mmol, 68% yield) as a yellow solid and **10** (17.8 mg, 0.320 mmol, 10% yield) as a yellow solid.

***tert*-Butyl 2-(9-bromo-6-oxo-6,7-dihydrobenzo[2,3]azepino[4,5-b]indol-5(12H)-yl)acetate (9).** **IR** (neat): 3286 (br s), 2982 (w), 2927 (w), 1735 (s), 1647 (s), 1450 (m), 1369 (s), 1303 (m), 1230 (m), 1149 (s), 1049 (w), 1022 (w), 956 (w), 902 (w) cm^{-1}; **^{1}H NMR** ($CDCl_3$, 400 MHz): δ 8.60 (s, 1H), 7.80 (s, 1H), 7.58 (d, *J* = 7.6 Hz, 1H), 7.43-7.42 (m, 2H), 7.35-7.26 (m, 3H), 4.43 (br s, 1H), 4.23 (br s, 1H), 3.93 (br s, 1H), 3.20 (br s, 1H), 1.41 (s, 9H) ; **^{13}C NMR** ($CDCl_3$, 100 MHz): δ 171.3, 168.6, 140.2, 136.0, 133.3, 128.8, 128.5, 126.5, 126.0, 125.7, 125.5, 123.9, 121.4, 113.4, 112.7, 110.6, 82.0, 53.7, 31.7, 27.9; **HRMS** (ESI-TOF) *m/z*: $[M + Na]^+$ Calcd for $C_{22}H_{21}N_2O_3NaBr$ 463.0633, Found 463.0633.

Di-*tert*-butyl 2,2'-(9-bromo-6-oxo-6,7-dihydrobenzo[2,3]azepino[4,5-b]indole-5,12-diyl)di-acetate (10). **IR** (neat): 2977 (w), 2981 (w), 1739 (s), 1674 (s), 1604 (w), 1496 (w), 1465 (m), 1369 (s), 1311 (w), 1284 (w), 1226 (m), 1159 (s), 1060 (w), 1026 (w), 991 (w), 952 (w) cm^{-1}; **^{1}H NMR** ($CDCl_3$, 400 MHz): δ 7.85 (s, 1H), 7.55-7.45 (m, 3H), 7.39-7.32 (m, 2H), 7.16 (d, *J* = 8.5 Hz, 1H), 4.88 (d, *J* = 17.6 Hz, 1H), 4.72 (d, *J* = 17.8 Hz, 1H), 4.45 (d, *J* = 16.9 Hz, 1H), 4.04 (d, *J* = 17.1 Hz, 1H), 3.90 (d, *J* = 14.1 Hz, 1H), 3.15 (d, *J* = 13.9 Hz, 1H), 1.47 (s, 9H), 1.44 (s, 9H); **^{13}C NMR** ($CDCl_3$, 100 MHz): δ 171.8, 168.6, 168.0, 141.3, 138.0, 135.1, 129.0, 128.1, 127.7, 126.1, 125.5, 124.3, 124.1, 121.6, 113.9, 112.1, 111.2, 83.0, 82.0, 77.2, 53.5, 47.8, 31.8, 27.9; **HRMS** (ESI-TOF) *m/z*: $[M + H]^+$ Calcd for $C_{28}H_{32}N_2O_5Br$ 555.1495, Found 555.1496.

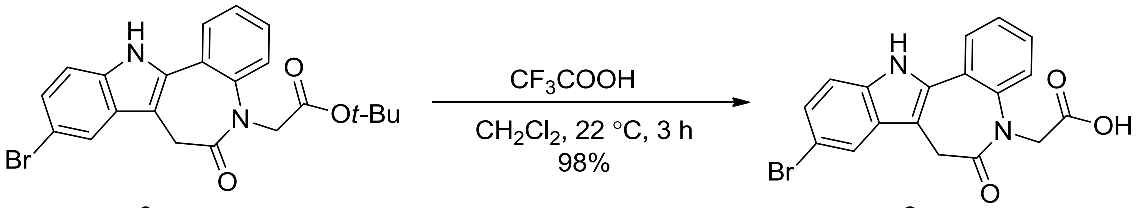

9 **8**

2-(9-Bromo-6-oxo-6,7-dihydrobenzo[2,3]azepino[4,5-b]indol-5(12H)-yl)acetic acid (8). A mixture of **9** (32.3 mg, 0.0732 mmol) and TFA(300 μL) in CH_2Cl_2 (700 μL) was allowed to stir at room temperature for 3 h. After that time, the resulting solution was concentrated in *vacuo.* The crude product was purified by recrystallization with diethyl ether to afford the desired product **8** (30.4 mg, 0.0789 mmol, >98%) as a white solid. **IR** (neat): 3500 (br s), 2923 (m), 2854 (w), 1677 (s),

1450 (m), 1392 (w), 1303 (w), 1207 (s), 1141 (s), 1026 (w) cm^{-1}; **^{1}H NMR** (DMSO-*d_6*, 400 MHz): δ 12.82 (br s, 1H), 11.92 (s, 1H), 7.93 (s, 1H), 7.72 (d, *J* = 7.8 Hz, 1H), 7.53-7.46 (m, 2H), 7.43-7.37 (m, 2H), 7.28 (dd, *J* = 8.5 Hz, 1.5 Hz, 1H), 4.30 (br s, 2H), 3.98 (br s, 1H), 3.06 (br s, 1H); **^{13}C NMR** (DMSO-*d_6*, 400 MHz): δ 170.9, 170.4, 140.0, 136.0, 134.0, 128.7, 128.0, 127.3, 125.3, 125.1, 124.7, 124.0, 120.7, 113.7, 111.8, 109.0, 52.6, 31.1; **HRMS** (ESI-TOF) *m/z*: $[M + Na]^+$ Calcd for $C_{18}H_{13}N_2O_3NaBr$ 407.0007, Found 407.0003.

- **1H NMR and 13C NMR Spectra**

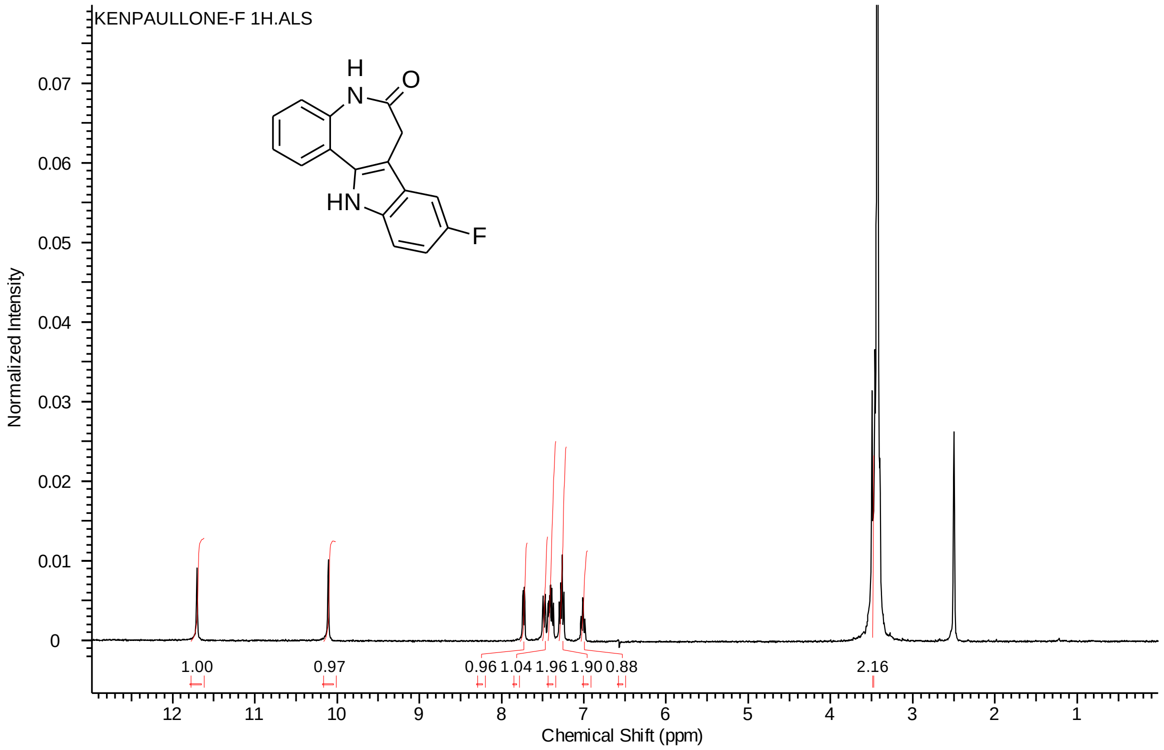

Figure SI 1. ^{1}H NMR spectrum of the compound **2**

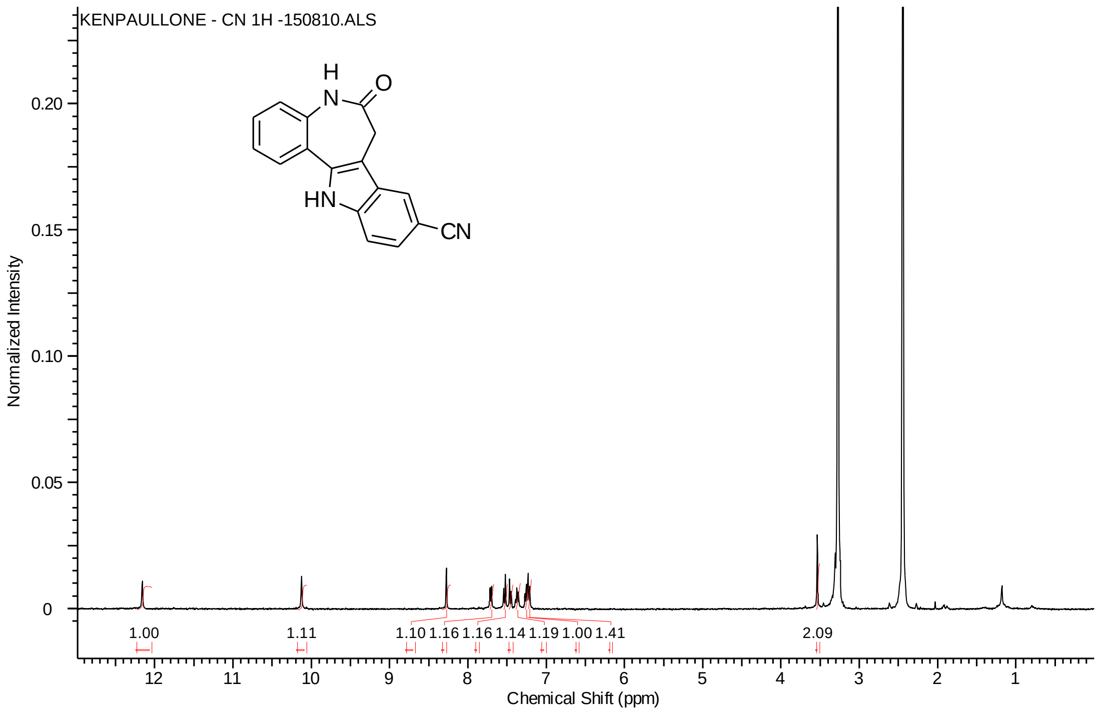

Figure SI 2. ^{1}H NMR spectrum of the compound **3**

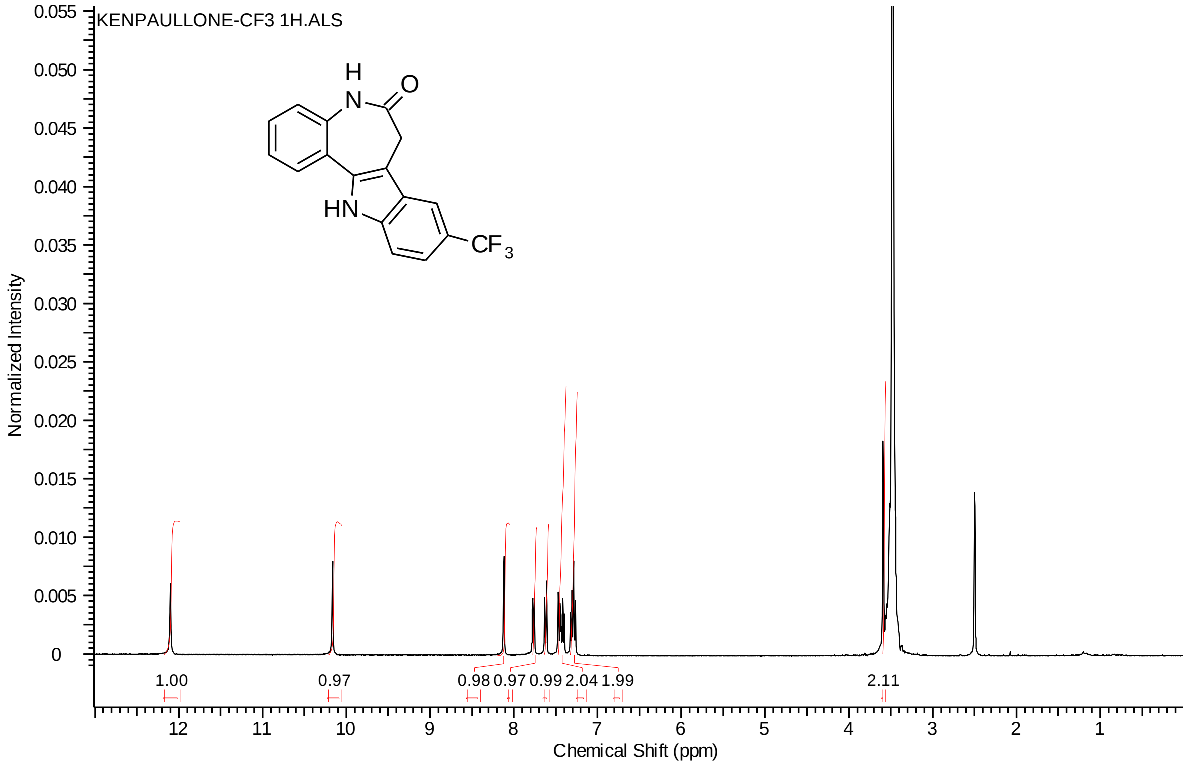

Figure SI 3. ^{1}H NMR spectrum of the compound **4**

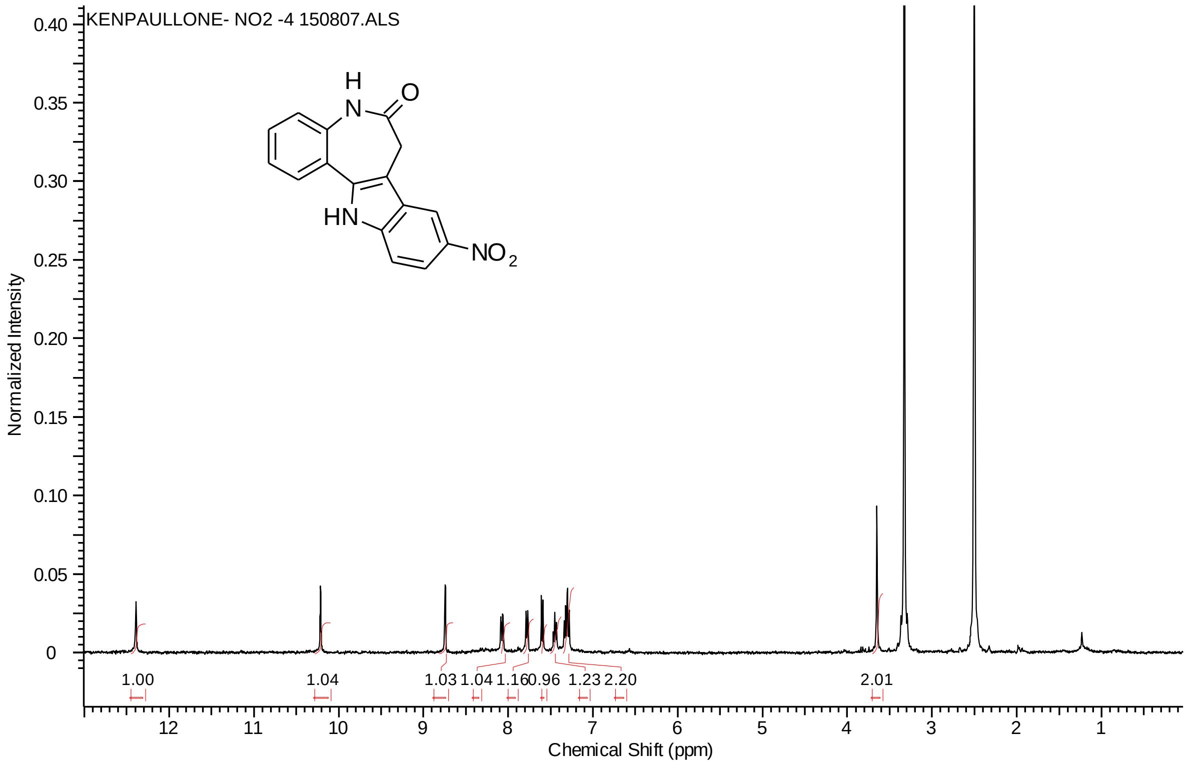

Figure SI 4. ^{1}H NMR spectrum of the compound **5**

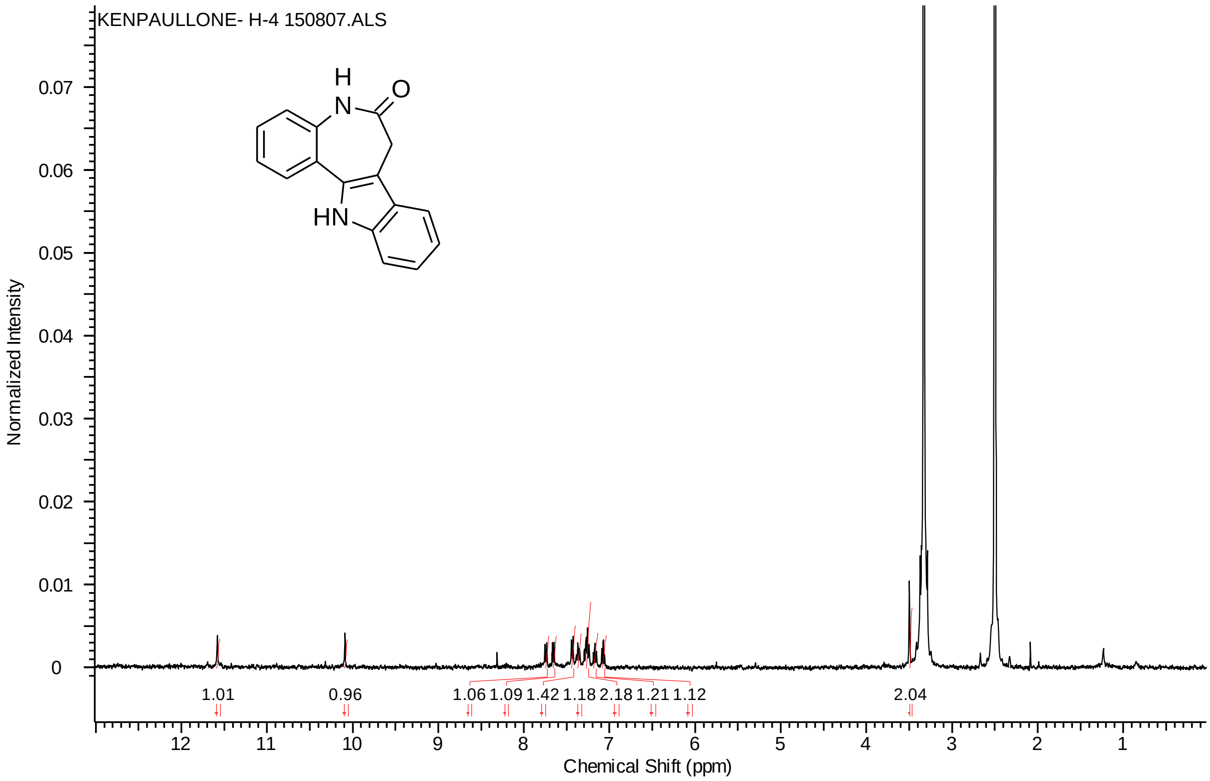

Figure SI 5. ^{1}H NMR spectrum of the compound **6**

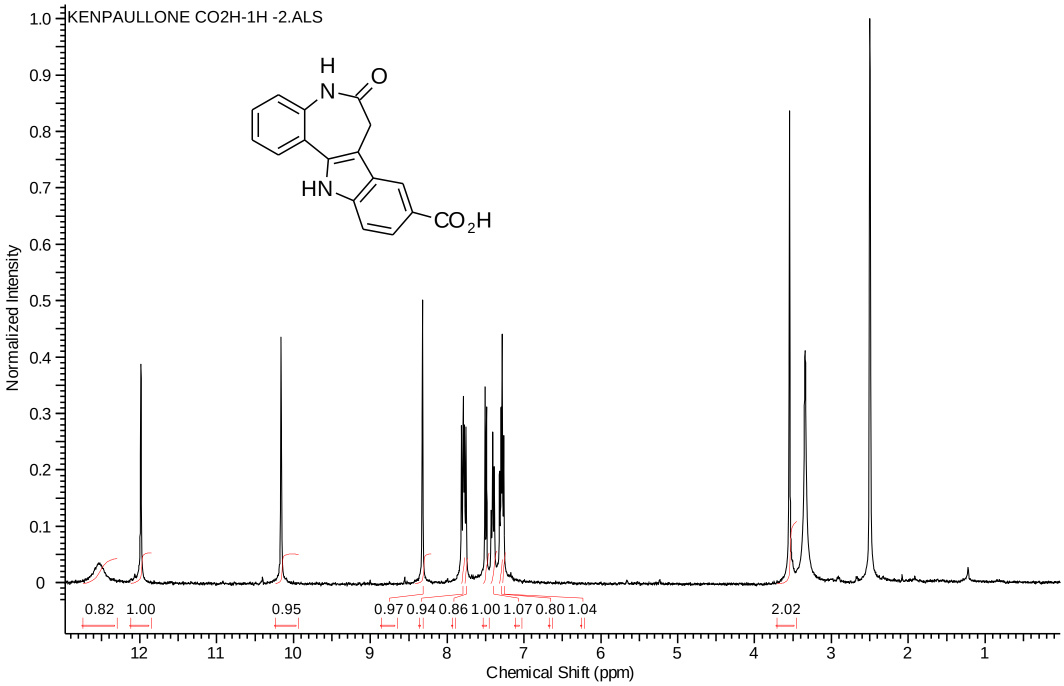

Figure SI 6. ^{1}H NMR spectrum of the compound **7**

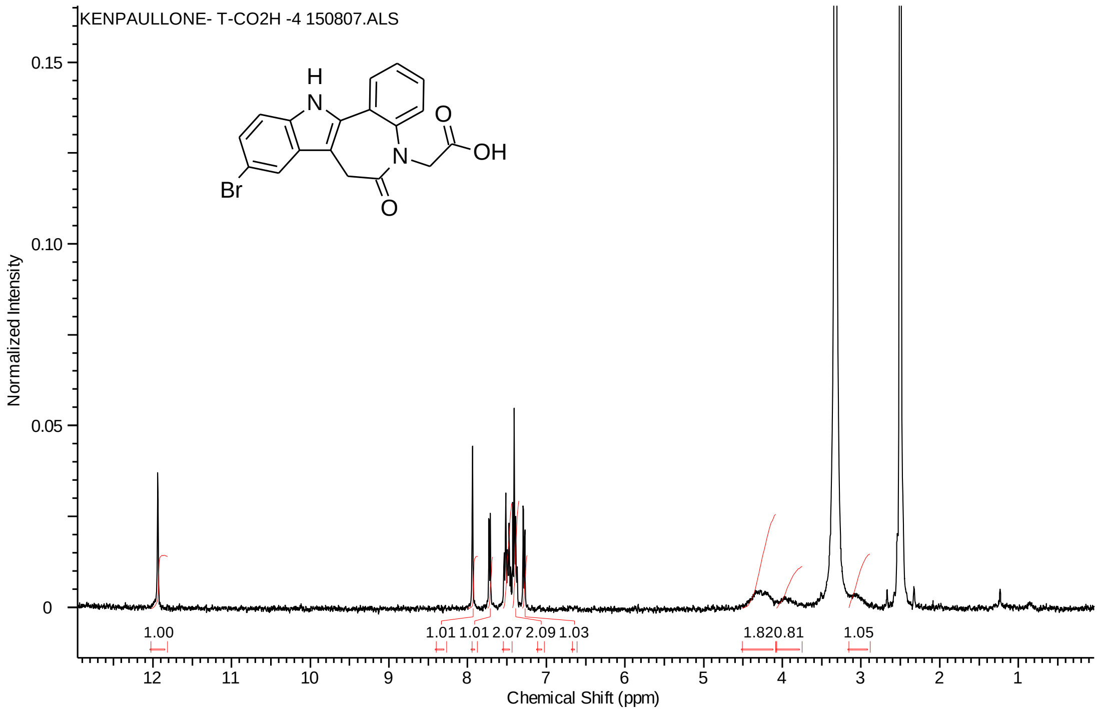

Figure SI 7. ^{1}H NMR spectrum of the compound **8**

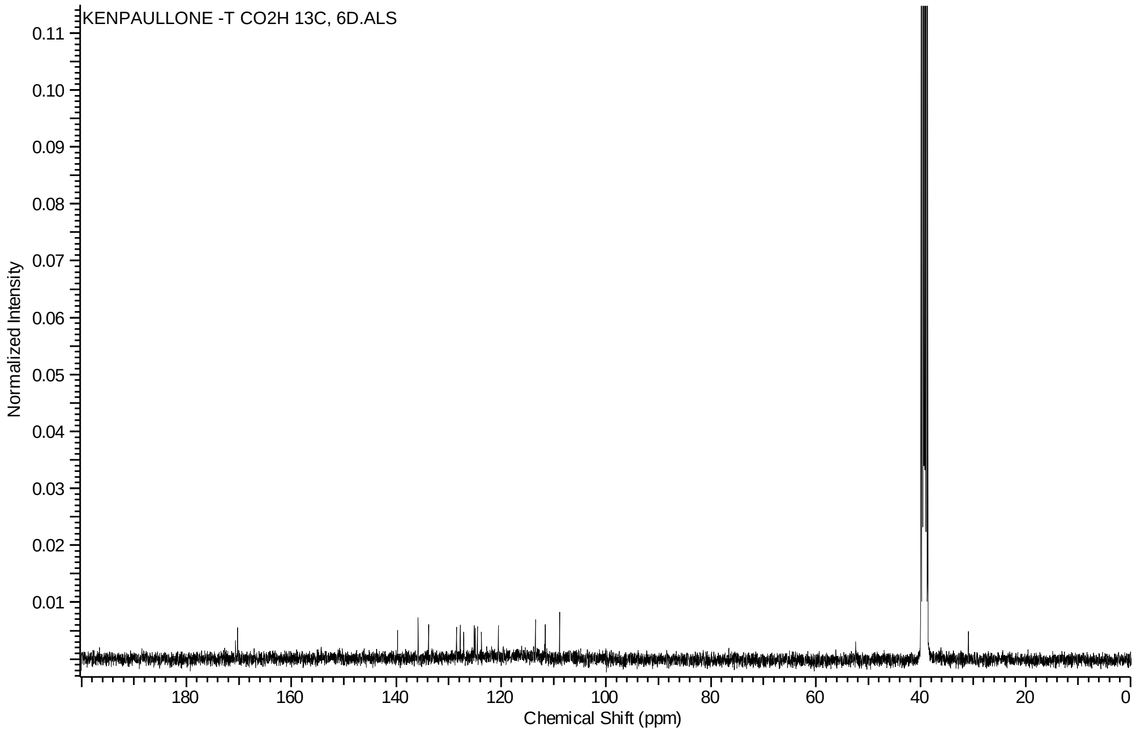

Figure SI 8. ^{13}C NMR spectrum of the compound **8**

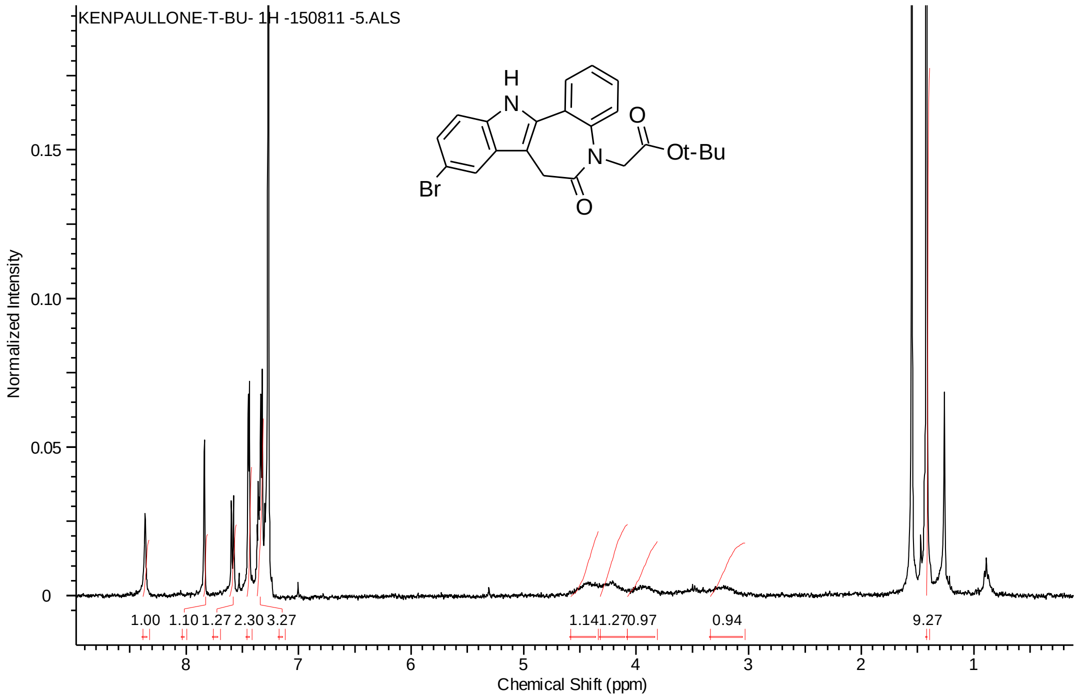

Figure SI 9. ^{1}H NMR spectrum of the compound **9**

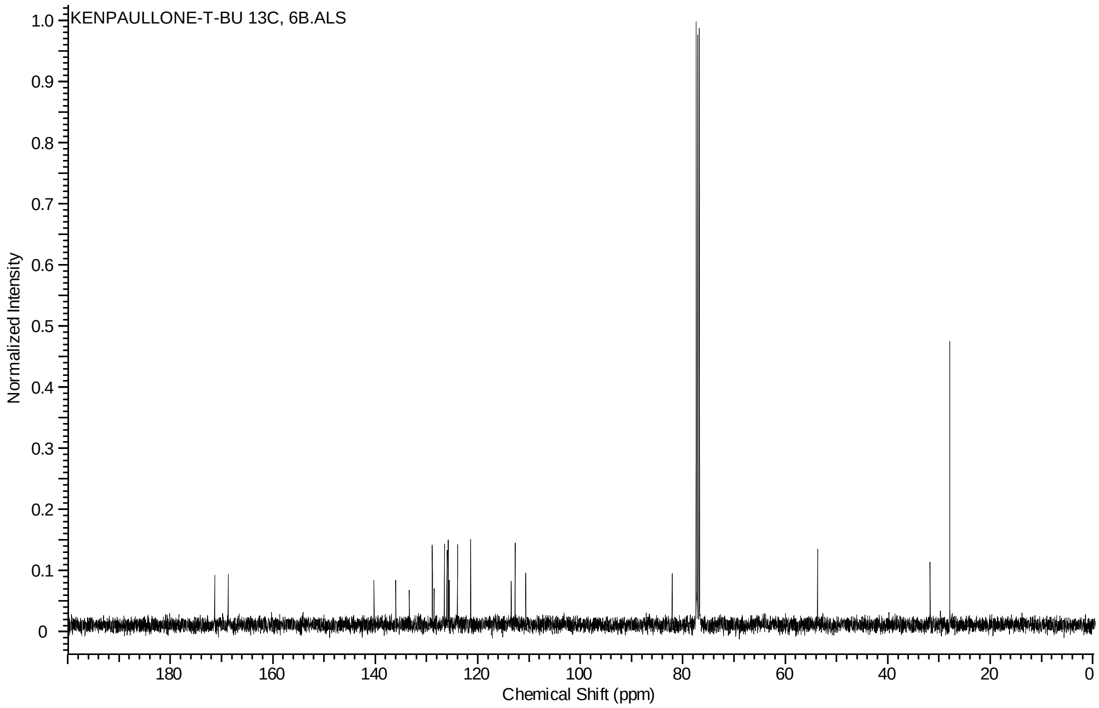

Figure SI 10. ^{13}C NMR spectrum of the compound **9**

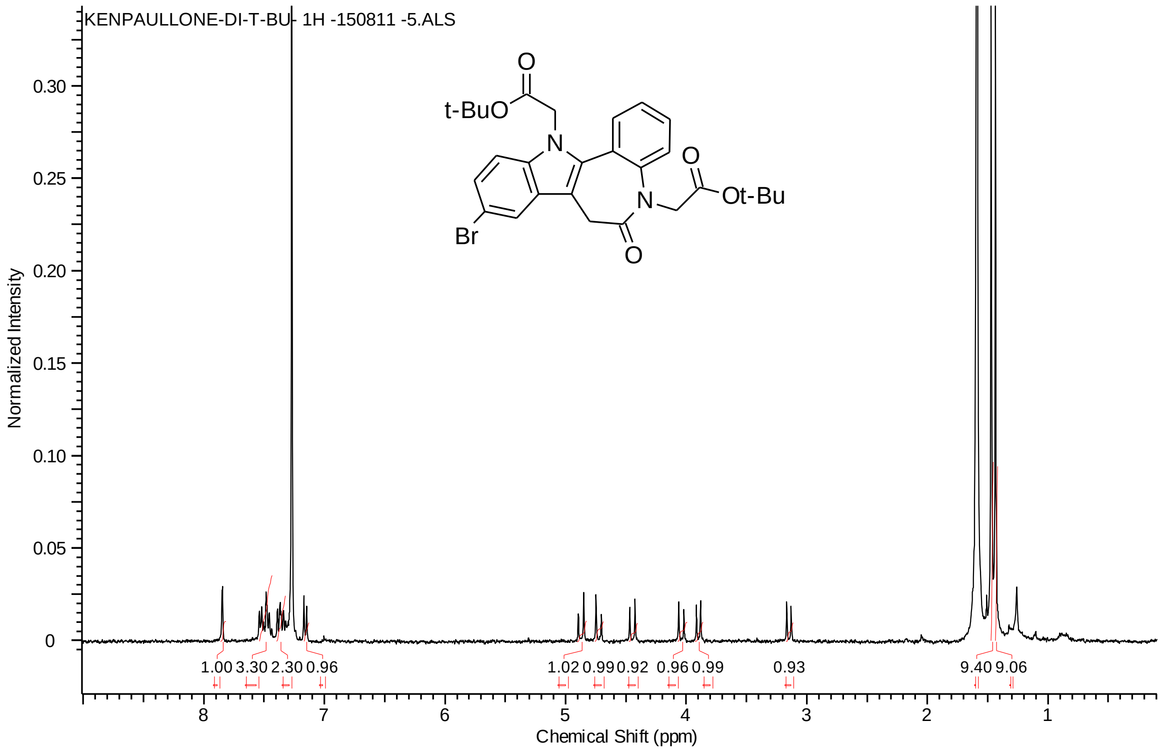

Figure SI 11. ^{1}H NMR spectrum of the compound **10**

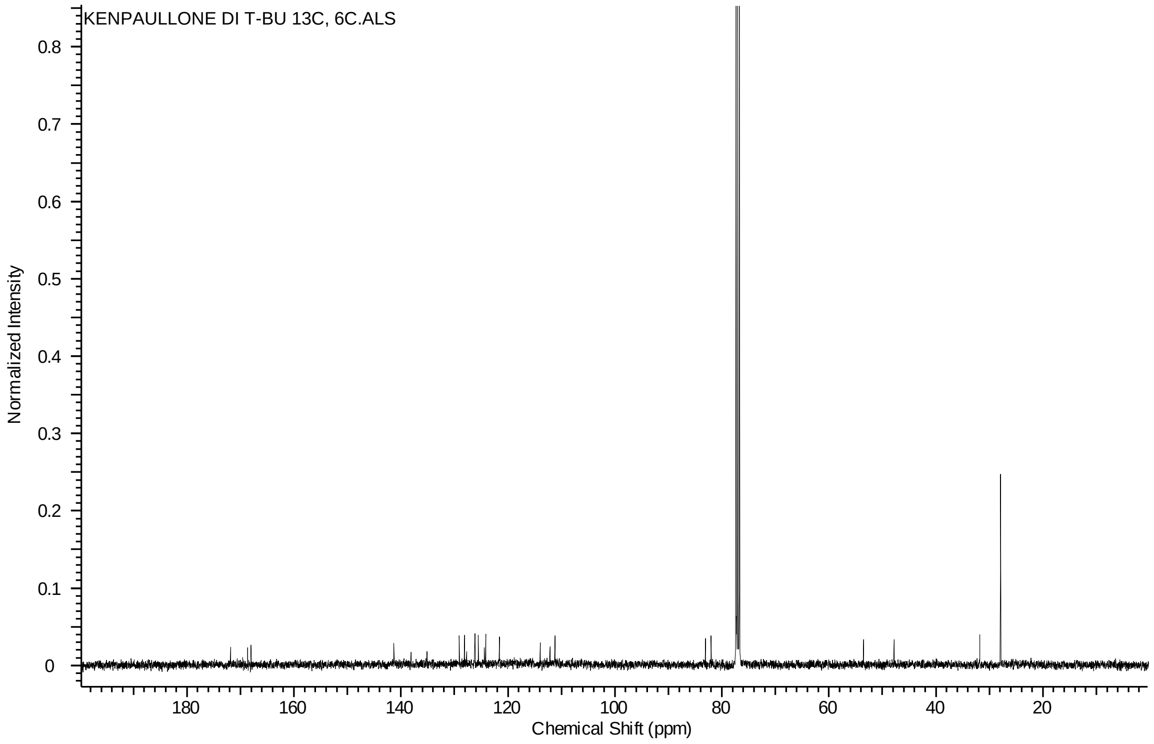

Figure SI 12. ^{13}C NMR spectrum of the compound **10**

References

[1] Duffey MO, Vos TJ, Adams R, Alley J, Anthony J, Barrett C, et al. Discovery of a potent and orally bioavailable benzolactam-derived inhibitor of Polo-like kinase 1 (MLN0905). J Med Chem. 2012;55:197-208.

[2] Schultz C, Link A, Leost M, Zaharevitz DW, Gussio R, Sausville EA, et al. Paullones, a series of cyclin-dependent kinase inhibitors: synthesis, evaluation of CDK1/cyclin B inhibition, and in vitro antitumor activity. J Med Chem. 1999;42:2909-19.
